# Liquid Chromatography Analysis of Common Nutritional Components, in Feed and Food

**DOI:** 10.3390/foods8010001

**Published:** 2018-12-20

**Authors:** Carolina Cortés-Herrera, Graciela Artavia, Astrid Leiva, Fabio Granados-Chinchilla

**Affiliations:** 1 Centro Nacional de Ciencia y Tecnología de Alimentos (CITA), Universidad de Costa Rica, Ciudad Universitaria Rodrigo Facio 11501-2060, Costa Rica; carolina.cortesherrera@ucr.ac.cr (C.C.-H.); graciela.artavia@ucr.ac.cr (G.A.); 2 Centro de Investigación en Nutrición Animal, Universidad de Costa Rica, Ciudad Universitaria Rodrigo 11501-2060, Costa Rica; astrid.leiva@ucr.ac.cr

**Keywords:** food and feed analysis, liquid chromatography, challenges, nutritional analysis, additives, contaminants

## Abstract

Food and feed laboratories share several similarities when facing the implementation of liquid-chromatographic analysis. Using the experience acquired over the years, through application chemistry in food and feed research, selected analytes of relevance for both areas were discussed. This review focused on the common obstacles and peculiarities that each analyte offers (during the sample treatment or the chromatographic separation) throughout the implementation of said methods. A brief description of the techniques which we considered to be more pertinent, commonly used to assay such analytes is provided, including approaches using commonly available detectors (especially in starter labs) as well as mass detection. This manuscript consists of three sections: feed analysis (as the start of the food chain); food destined for human consumption determinations (the end of the food chain); and finally, assays shared by either matrices or laboratories. Analytes discussed consist of both those considered undesirable substances, contaminants, additives, and those related to nutritional quality. Our review is comprised of the examination of polyphenols, capsaicinoids, theobromine and caffeine, cholesterol, mycotoxins, antibiotics, amino acids, triphenylmethane dyes, nitrates/nitrites, ethanol soluble carbohydrates/sugars, organic acids, carotenoids, hydro and liposoluble vitamins. All analytes are currently assayed in our laboratories.

## 1. Introduction

Food and feed analysis are paramount to assess both nutritional quality and safety of commodities. Interconnectivity of food sources [1,2] and new processing techniques [3] make for a more diverse and complex food supply. Legal thresholds have been stipulated that establish acceptable levels for individual chemical additives, residues, and contaminants in products [4,5]. Feed is a paramount target for analysis since it situates at the start of the food chain and poor feed quality can affect the yield on food-producing animals [6]. Understanding the complexities of food safety is the goal of approaches such as One Health [7], Farm-to-Fork [6], or MyToolbox [8]. Furthermore, feed contaminants carryover downstream can reach products such as meat, eggs, and milk (see for example the transference of aflatoxin M_1_ from aflatoxin B_1_-contaminated feed). Ingredients either destined for food or feed production (e.g., cereals) are among the fundamental constituents for several staple commodities. Other regulations require food and feed labeling to list ingredients relating to the nutritional content [9,10]. All stakeholders involved in the food and feed chain must be able to assess product quality and safety. Hence, it is imperative to rely on techniques that meet several analytical performance parameters. More and more, food and feed analysis methods are based on LC (liquid-chromatography) [11,12], which has proven to be an optimal technology for screening, detection, and quantification of a vast variety of analytes (see Table 1). The reason behind this is related to the molecular affinity between the analyte and also: i. the mobile phase (which is usually a mixture of solvents) ii. stationary phase (modified silica and polymer scaffolds). Within the LC approach itself, several alternatives are available for a researcher to resolve a specific task at hand. Each analyte presents its own unique trials.

To successfully analyze or isolate a compound, a researcher is faced with several questions: What is the problem to solve, the objective or purpose for the analysis? Is the required data qualitative or quantitative? Are there two or multiple compounds to be separated? What are the physicochemical characteristics of the target(s)? What matrix was the analyte recovered from and which interferences are expected? What is the amount of analyte expected to be recovered? What equipment is accessible in the laboratory?

Considering the above, a suitable column (Table 2) and detection system must be selected (Table 3). Sample preparation can aid to solve some of these issues, especially those regarding interferences and sensitivity but cannot solve issues with poor detector choice. For example, if sensitivity is a problem using the selected detection system on hand and no other system is available, the initial sample mass can be increased, or a concentration step (evaporation or solid phase extraction (SPE)) can be performed. Additionally, the sample injection volume can be expanded to improve sensitivity.

Additionally, automation is relevant for conserving resources and reducing turnover times. An analyst can program an autosampler to increasingly adjust the volume of a standard with a fixed concentration. For example, to construct a calibration curve between 1000 and 62.5 µg L^−1^, one could use a 1000 µg L^−1^ standard and instruct the sampler to take from the same vial 20 µL, 10 µL, 5 µL, 2.5 µL, 1.25 µL, consecutively. The sampler will construct a calibration curve without analyst intervention and this automation will reduce errors. Autosamplers are designed to inject small volumes without significant loss, with good precision, and adequate reproducibility. They can also inject variable amounts, dilute the sample prior to injection and perform precolumn derivatization [91]. If a sample is outside of calibration standard of higher concentration, an analyst can inject a different volume to ensure it will fit among the calibration curve range. However, injection volume has an impact on peak shape. The method must be validated to show this is a valid approach. (See for example, [92]). Reference for one example of the versatility of an LC system and capabilities for its automation. In this review, we intend to give the reader a thorough background on the common analyses performed, for quality assurance and safety, in food and feed laboratories. We will include the most recent and relevant experience gathered for each test while pointing out the difficulties that each essay presents and the common ground shared by both types of laboratories.

## 2. Measurements of Commonly Consumed Food Commodities

### 2.1. Polyphenols

**Figure 1:**
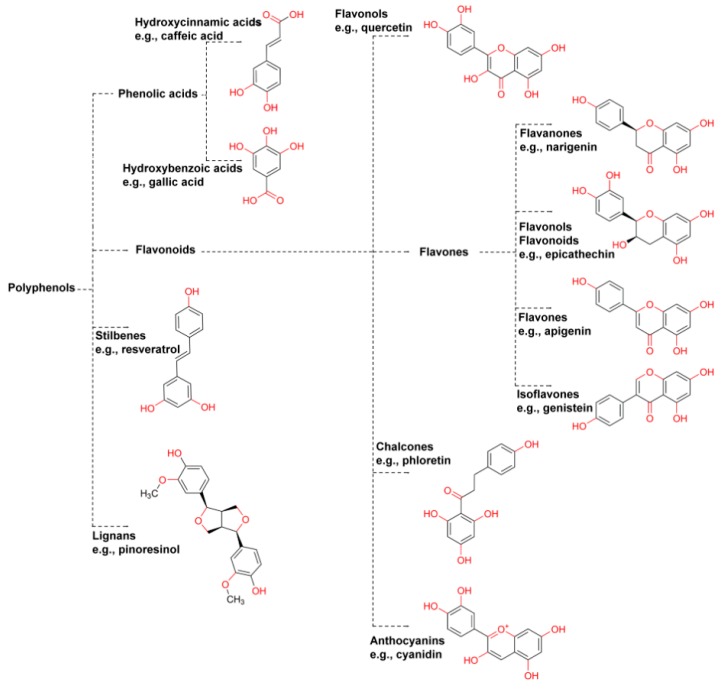
Polyphenols structure and classification [97]. Highly functionalized structures account for the molecules radical scavenging, metal ion chelating, and enzyme inhibition. Hydrogen bonding can stabilize phenoxyl radicals.

Polyphenols usually refers to several chemical compounds including flavanols (e.g., catechins and tannins from tea), flavanones (i.e., hesperidin from citrus fruits), flavonols (e.g., quercetin from tea, apples and onions), “chlorogenic acids” (including hydroxycinnamic acids caffeic, ferulic, *p*-coumarinic acids usually extracted from coffee), anthocyanins (which are partly responsible for imparting color to plant structures), and stilbenes (e.g., from berries, grape skins and peanuts) (Figure 1) [93].

These compounds, secondary metabolites from plants [94], have, among other functions, a protective capability within the vegetable tissue, structure, and support [94], and, even, pollinator attraction [95]. For example, chlorogenic acid (i.e., the esterification product between caffeic and quinic acid) is an intermediate in lignin biosynthesis [96]. Data suggests that long-term consumption of such compounds can have beneficial effects [94] as it can improve an organism’s antioxidant capacity [93] which in turn relates, for example, to cognitive improvement [97] and reduction in adipogenesis and oxidative stress [98]. Fruits, especially berries, are [97,98,99,100,101] rich in these bioactive compounds, both extractable [102,103] and non-extractable [104]. From the technological standpoint, polyphenol safeguard is paramount to achieve functional foods [105] with added value (e.g., beverages) and a bioactive capacity of compounds as close as those from the raw material. Several operation units have been applied to fruits to assess polyphenol retention after processing including nanofiltration [101], high hydrostatic pressure [106], and drying [107,108]. Method-wise, the solvent has a profound effect on the number and type of polyphenols extracted. Polyphenol analysis must first identify the type of matrix to be analyzed, the chemical nature of the polyphenols of interest, and different solvents and solvent systems should be examined. The most appropriate solvent for the case in hand (i.e., maximizing compound diversity and yield) should be the one selected [109]. For example, Flores and coworkers resuspended the methanolic extract in hexane, chloroform, ethyl acetate, and *n*-butanol and reanalyzed each fraction. Ethyl acetate fraction exhibited the best results [110]. Finally, though polyphenols are usually related to health applications [111,112], antinutritional effects should be considered [109]. Some examples of polyphenol analysis are included in Table 4.

Gordon and coworkers used accelerated solvent extraction (ASE) to characterize polyphenolic compounds in *Psidium guineense* Sw., *Syzygium cumini* (L.) Skeels, *Byrsomina crassifolia* (L.) Kunth, and *Pouteria macrophylla* (Lam.) Eyma. [113]. ASE techniques allow for multiple extractions simultaneously. Swifter assays are obtained which, in turn, expedite research results and minimize solvent waste [114] when compared to common extraction methods (e.g., Soxhlet, sonication). Anton and coworkers investigated the effect of ripening in tomato polyphenols content and antioxidant capability. A differential mass spectrometry approach allowed the authors to conclude that cultivar-dependent patterns are observed during ripening (e.g., maximum concentrations of polyphenols achieved half-ripe stage) [115]. Radovanović and coworkers, associated polyphenols from berries to antibacterial activity [116]. Veljković and coworkers analyzed phenolic compounds in different types of tea. Nettle/pineapple, and bearberry/raspberry teas showed the lowest and highest phenolic contents, respectively [117]. Miletić assessed polyphenols in dried and candied fruit. In this particular case, acid hydrolysis was applied to the previously dispersed methanolic extracts to free matrix-bound polyphenols [118]. One g *tert*-butyl hydroquinone/100 mL was added during extraction as a radical sink to protect polyphenols. Kowalska and coworkers used preparative chromatography to remove non-phenolics [98].

Tentative screening for *Psidium friedrichsthalianum* (Berg) Niedenzu pulp showed 1,5-dimethyl citrate, 1-*trans*-cinnamoyl-β-d-glucopyranoside, sinapic aldehyde-4-*O*-β-d-glucopyranoside, 1,3-*O*-diferuloylglycerol, and 3,3′,4-tri-*O*-methylellagic acid-4′-*O*-d-glucopyranoside [110]. Phenolic compounds from pink guava from Costa Rica have been recently reported, *n* = 60 phenolic compounds were characterized. The authors report for the first time in *P. guajava n* = 42 compounds in the fruit’s peel and flesh, and *n* = 24 new compounds, e.g., phlorizin, nothofagin, astringin, chrysin-C-glucoside, valoneic acid bilactone, cinnamoyl-glucoside, and two dimethoxy cinnamoyl-hexosides [119]. During polyphenol analysis, HLB^®^ SPE (Hydrophilic-Lipophilic, Balance Solid Phase Extraction) cartridges are used routinely for clean-up. At least one research group has applied this approach to assay polyphenols and vitamin C in plant-derived materials [121]. Interestingly, when using the Folin–Ciocalteu spectrophotometric approach, ascorbate is considered interference and must be eliminated from the eluate (usually taking advantage of ascorbate thermolability) or else the measurements are overestimated. However, simultaneous retention of both analytes in the SPE cartridge can be exploited, if HPLC methods are used instead. We recommend that in countries in which fruits with high polyphenol content are readily available (and in considerable quantities), preparative separation of polyphenol fractions is a possibility for obtaining pure compounds (See for example, [122]). Finally, vanillic acid was reported in cocoa pod polyphenol-rich extracts. Interestingly, the application of 2000 mg L^−1^ of this cocoa extract to a vegetable oil improved its oxidative stability and shelf-life [123].

#### Method Application Experience

In our laboratory, ultrasound-assisted extraction is preferred for reducing processing time and avoiding degradation of the compounds. Additionally, polyphenols are quite light sensitive, hence yellow lights are used during the extraction using acetone-water or methanol-water solutions. As the polyphenol family is extensive and chemically diverse, a surface response design is always recommended to assess the appropriateness of the solvent system (i.e., selecting a solvent that provides the highest yields). Samples with a high lipid content (i.e., > 5g total fat/100 g) usually cause significant interferences and must be defatted previous to polyphenol extraction. It is usual to add additional antioxidants (e.g., ascorbic acid) to polyphenol extracts to protect them from oxidation. Finally, it is common to find natural existing polyphenols as adducts with protein or carbohydrate moieties. These adducts are usually formed by non-covalent interactions (e.g., salt bridges); therefore, by adjusting the extract ionic strength, one can remove these artifacts. Sugar adducts are considerably more difficult to analyze since only a few compounds are commercially available (e.g., cyanidin 3-*O*-glucoside chloride). Hydrolysis (mild acidic, basic or enzymatic) is the usual approach to circumvent the lack of these commercial standards. Availability of mass spectrometry or nuclear magnetic resonance (NMR) can help elucidate unknown compounds and adducts.

### 2.2. Capsaicinoids

**Figure 2:**
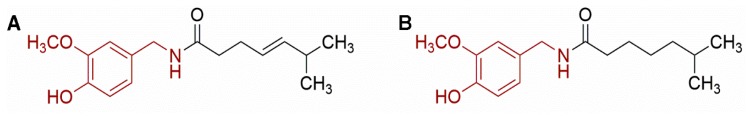
Chemical structures for (**A**) capsaicin (8-methyl-*N*-vanillylamide) and (**B**) dihydrocapsaicin (8-methyl-*N*-vanillylnonamide), the aromatic vanillyl radical is shown in red.

Capsaicinoids are plant metabolites from the *Capsicum* genus which give pungency to chili peppers [124]. Scoville scale which measures the spiciness of the fruits (originally, tested by sensory assays) is reported in function of capsaicin concentration (i.e., mg capsaicin kg^−1^ × 16 [125]). Today, the most reliable, rapid, and efficient method to identify and quantify capsaicinoids is HPLC. Measurement of this molecules is significant as a quality measure of chili pepper (22 domesticated varieties consumed regularly worldwide), a crop which is of significant cultural and global trade market value [126]. More than 20 different capsaicinoids have been described; the foremost capsaicinoids found in these plant structures include capsaicin and dihydrocapsaicin [127] (Figure 2).

#### 2.2.1. Measurement of Capsaicin and Dehydrocapsaicin in Real Samples

Research reports have described capsaicinoid analysis; the most recent are summarized in Table 5. Garcés-Claver and coworkers determined capsaicin and dihydrocapsaicin in two different scenarios, i.e., fruits grown in summer and then in spring [128]. The authors concluded that capsaicinoids varied largely among fruit families and that these families did not respond similarly to producing these capsaicinoids when their fruits were grown in the two seasons tested [128].

Goll and coworkers optimized a cyclic solid support free liquid–liquid partition to separate a capsaicin and dehydrocapsaicin mixture into two sequentially collected product streams. This approach may serve as a base for compound purification before chemical characterization. With this optimization, the authors demonstrated theoretical and predictive tools are useful in preparative chemistry and process design [129].

The pretreatment of capsaicinoid determination (i.e., extraction steps) is usually straightforward, and the majority of methods are based on methanol-based extraction. However, Lu and coworkers reviewed several techniques that can be used to extract capsaicinoids successfully [136]. Ma and coworkers [131] used capsaicin and dihydrocapsaicin, and nonivamide [132] were selected as adulteration markers to authenticate vegetable oils. No capsaicinoid compounds were found in edible vegetable oils, thereby ruling out a possible adulteration source. The authors prepared immunosorbents by covalently coupling highly specific capsaicinoid polyclonal antibodies with CNBr-activated Sepharose 4B and packed into a polyethylene column [131]. This research is interesting, from the clean-up standpoint, since the authors adjusted the major parameters affecting the immunoaffinity column extraction efficiency (i.e., loading, washing, and eluting conditions) [131]. Schmidt and coworkers compared different chili peppers available in Austria and compared their contents of capsaicin and dihydrocapsaicin [133]. The authors used UPLC (Ultra-Performance Liquid-Chromatography) and hence obtained a reduced resolved chromatogram for both compounds of just 1.7 min. [133]. The authors also corroborated that the highest capsaicinoids content was in the fruits’ placenta and the seeds. Similarly, Sganzerla and coworkers obtained a complete separation under 4 min [134]. The above examples correspond to high-throughput methods of analysis.

Finally, ingested capsaicinoids can persist in the bloodstream and can be determined in plasma using LC coupled with tandem mass spectrometry [137]. Intestinal absorption and metabolisms (via capsaicinoid glucuronides) have also been reported for a mammal [138]. At the same time, dietary capsaicin has been linked to the browning of adipose tissue, which in turn, promotes energy expenditure [139].

#### 2.2.2. Method Application Experience

As shown, capsaicinoids can very well be measured by using a wavelength in the 200–400 nm UV range. However, fluorescence analysis can be performed (λ_ex_ 280 nm λ_em_ 338 nm) improving sensitivity dramatically [134], an approach preferred by our laboratory for routine analysis. A short column with a smaller particle size seems to improve both resolution and sensitivity.

### 2.3. Caffeine and Theobromine

**Figure 3:**
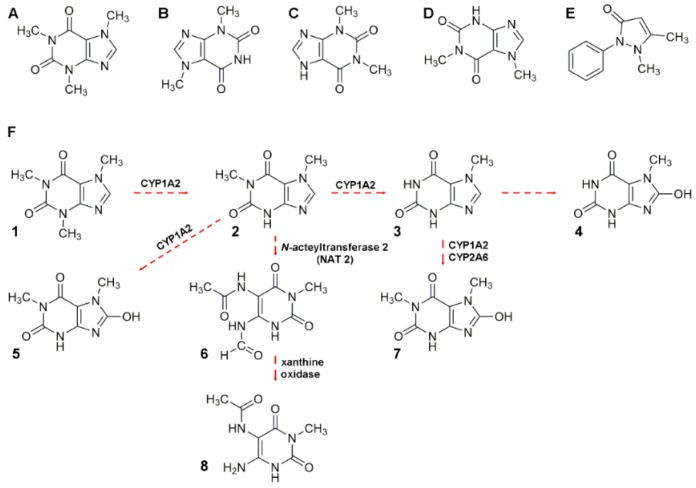
Chemical structures for (**A**) caffeine (1,3,7-trimethylxanthine), (**B**) theobromine (3,7-dimethylxanthine), (**C**) theophylline (1,3-dimethylxanthine), (**D**) paraxanthine (1,7-dimethylxanthine), and (**E**) antipyrine (2,3-Dimethyl-1-phenyl-3-pyrazoline-5-one or phenazone). (**F**) Caffeine biotransformation pathway is dependent on the CYP1A2 and CYP2A6 enzyme system. **1**. 1,3,7-trimethylxanthine **2**. 1,7-dimethylxanthine **3**. 7-methylxanthine **4**. 7-methyluric acid **5**. 1-mthyluric acid **6**. 5-acetylamino-6-formylamino-3-methyluracil **7**. 1,7-dimethyluric acid **8**. 5-acetylamino-6-amino-3-methyluracil [145].

Caffeine and theobromine are naturally occurring methylxanthines with antioxidant potential [140] (Figure 3). There are some misconceptions regarding health effects caused by caffeine ingestion [140]. On the contrary, theobromine (and cocoa) consumption has demonstrated beneficial effects [141]. Coffee, cocoa, tea, and caffeine-containing beverages (e.g., soft and energy drinks) are widespread and relevant food commodities. For example, caffeine intake has been calculated at 25 and 50 mg per day for children and adolescents aged 2–11 and 12–17 years, respectively. The more relevant caffeine sources were soda and tea as well as flavored dairy (for children aged < 12 years) and coffee (for those aged 12 years and above). Similarly, caffeine consumption has been between 2.5–3 and 400 mg kg^−1^ bw (body weight) day^−1^ for children and adults, respectively [142,143]. The evidence is suggesting an alimentary impact as some nutrients are poorly absorbed when combined with alkaloids [140]. Caffeine analysis is common in the food industry (e.g., quality control in beverages) and research (e.g., alkaloid carrying plants); it has also been incorporated in academia and student curricula [144].

#### 2.3.1. Alkaloid Analysis and Reported Application to Real Samples

Several methods have been developed for alkaloid analysis in food samples. Also, methods for studying the fate of these alkaloids have been documented (Table 6). For example, Grujić-Letić and coworkers, analyzed 12 commercial tea and coffee products, non-alcoholic energy drinks and foods (including mate, green tea, and black tea), 5 combined preparations of over the counter non-steroid anti-inflammatories and water samples collected from 7 representative locations of the Danube River [146]. This paper represents a clear example of method versatility, as a single analyte was recovered, from variable matrices, and assessed using a similar procedure. This analysis was not only used for characterization, but also demonstrated a potential for quality control in commercial products (e.g., compliance of the nutritional label) and water. In water samples, the highest caffeine concentration found was 306.120 ± 0.082 ng L^−1^ during springtime. Gonçalves and coworkers recently demonstrated that caffeine might be a suitable chemical marker of domestic wastewater contamination in surface waters [147].

Shrestha and coworkers developed a method for use as quality control. Concentrations of Nepalese tea and coffee ranged from 1.10 to 4.30 mg caffeine kg^−1^ dry basis [156]. Fajara and Susanti also determined caffeine in coffee beverages; they found 109.7–147.7 mg caffeine kg^−1^ per serving [157]. Gliszczyńska-Świgło and Rybicka used both a photodiode and fluorescence detector to monitor both caffeine and water-soluble vitamins, simultaneously, in energy drinks [148]. Aşçı and coworkers analyzed caffeine in soft drinks [158]. The authors used Behnken response surface design to optimize HPLC conditions. Optimized variables included pH, 6.0, flow rate, 1.0 mL min^−1^ and a mobile phase ratio, 95% [158]. Similarly, preservatives sorbate and benzoate also can be determined with caffeine simultaneously in sports drinks [149]. Ortega and coworkers compared data from HPLC- and UPLC-MS/MS (MS/MS also known as tandem mass spectrometry). The authors analyzed procyanidin oligomers (mono to nonamers) and catechin, epicatechin, caffeine, theobromine. The analysis was performed under 12.5 min [150]. Recently, Rodríguez-Carrasco and coworkers used to analyze polyphenols and alkaloids in cocoa-based products. Mainly, they compared three different coffee varieties including “Forastero”, “Trinitario”, and “Criollo”. Mostly, theobromine was found in major quantities relative to caffeine except Criollo 70 and 75% where the theobromine/caffeine ratio is ca. 1:1. Of all samples examined, Criollo varieties showed the highest quantities of alkaloids. [151]. Interestingly, a positive association has been described between cacao polyphenol absorption and theobromine [159]. Other identifying markers, such as fatty acids, have also been reported as tools for discrimination among coffee varieties. The authors were able to discern *Coffea arabica* (Arabica) and *Coffea canephora* (Robusta) using ∑MUFA, 18:3n3, ∑MUFA/∑SFA [160].

#### 2.3.2. Alkaloid Bioavailability and Transference to Biological Samples

Caffeine is rapidly absorbed following oral consumption; maximum blood (plasma) levels are usually reached within 30 min [140]. Caffeine bioavailability studies have been performed in human plasma, for example, Alvi and Hummami monitored caffeine and antipyrine (Figure 3). Caffeine in human plasma was stable for at least 24 h at room temperature or 12 weeks at –20 °C [153]. Caffeine is a demonstrated therapeutic agent for apnea of prematurity. Hence, López-Sánchez developed a method to monitor caffeine in serum to demonstrate that the drug had achieved its therapeutic levels (i.e., 30 or 35 μg mL^−1^) [161]. Cleanup using SPE adapted in multiple well plates, as the one used in the former study, is an easy way to process several samples simultaneously, instead of the one-on-one cartridge approach. Only in 85% and 78% of the cases studied, maternal and newborn absorption of caffeine was demonstrated, respectively. Another research group investigated caffeine metabolism based on CYP1A2 enzyme activity. The presence and ratio of theophylline, paraxanthine, theobromine, and caffeine (Figure 3) was evaluated in human saliva [154]. The authors collected saliva of healthy subjects after consumption of a caffeinated beverage and obtained data of compared chromatographic profiles from the saliva of smoking (active xenobiotic hepatic metabolism) and non-smoking subjects [154]. Saliva, plasma, and urine already have been demonstrated valuable to intervention studies for cocoa [155,162]. Kobayashi used differential chromatogram analysis to narrow the signal width for caffeine, in urine samples, to improve separation demonstrating that peak enhancing posterior to injection is possible [163]. Finally, Ramdani and co-workers incorporated green and black tea powder into bovine diets demonstrating that alkaloids, catechins, and theaflavins diminished ammonia and methane productions without any detrimental effect on rumen functions *in vitro* [51].

Although theobromine is not a usual analyte for feed analysis, is noteworthy that the 2002/EC/32 regulation sets limits for the analyte at 300 mg kg^−1^ for compound feed, except for adult cattle feed, where the threshold is laxer (i.e., 700 mg kg^−1^).

#### 2.3.3. Method Application Experience

Tea and coffee sample extracts are rich in tannins and other non-desired compounds that may generate matrix effects and reduce the shelf life of an analytical column. We have successfully used MgO to remove said interferences while increasing the extract pH. An alkaline medium ensures positively charged alkaloid molecules. Furthermore, defatting is vital for an adequate recovery when a lipid-rich sample is treated (e.g., cacao seeds, >30 g total fat/100 g), especially, if aqueous extracting is employed. We suggest the use of efficient organic solvents; *n*-hexane, petroleum benzine, for example, have been exploited. Minimal amounts possible should be used, as this otherwise generates waste. Chlorinated solvents and ethyl ether should be avoided, as alkaloids exhibit some degree of solubility in these solvents which, in turn, may affect recovery.

### 2.4. Cholesterol

Cholesterol ((3*S*,8*S*,9*S*,10*R*,13*R*,14*S*,17*R*)-10,13-dimethyl-17-[(2*R*)-6-methylheptan-2-yl]2,3,4,7,8, 9,11,12,14,15,16,17-dodecahydro-1*H*-cyclopenta[a]phenanthren-3-ol), is a waxy steroid metabolite found in the cell membranes and transported in the blood plasma of all animals [164]. This sterol plays a role in metabolic (e.g., precursor for bile acids and steroid hormones) and structural processes (e.g., regulates biological membrane fluidity) [165,166]. Cholesterol can be introduced to the metabolism through *de novo* synthesis or diet [162]. In plant structures, similar compounds are found such as phytosterols and stanols [167]. However, when analyzing cholesterol, one must consider that the amount of cholesterol made by many plants is not negligible [168]. Nutritional information regarding cholesterol content in food and intake through dietary sources is relevant, as overload can drastically increase plasma cholesterol levels and, hence, health risks. From a methodological standpoint, a considerable advantage in using the LC approach is that lipid oxidation is negligible, as measurements can be performed at relatively low temperatures. Herein are detailed some examples of cholesterol analysis in food samples (Table 7).

Albuquerque and coworkers compared both HPLC and UPLC for the analysis of eggs, egg yolks, sour cream, and chicken nuggets. The latter approach rendered a method with 8-fold less solvent waste and ca. 4-fold more sensitivity, with a decreased analysis time (i.e., 4 min) [166]. The initial sample mass used from the assay was optimized; 0.25 g and 1 g for samples with relative lower (e.g., sour cream) and higher (e.g., egg yolk) cholesterol contents. The authors also compared different cooking methods for the chicken nuggets (baked vs. deep frying). They found that cholesterol content was higher in the oven baked goods. This is a result of the processing as the meat loses water during baking. Meanwhile, water/oil exchange occurs during frying. Although several solvents were tested, the authors concluded that an acetonitrile/2-propanol solvent system was the most successful in eluting the cholesterol molecule [166]. Cholesterol analysis usually renders clean chromatograms since most interferences are eliminated by saponification. Saponification segregates the molecule of interest from the saponifiable lipid fraction (e.g., acylglycerols) and hydrolyzes cholesterol esters. This step has been considered critical for cholesterol analysis in food matrices [166]. Furthermore, Cruz and coworkers, quantitatively, compared several extraction methods on freeze dried and thawed seafood samples [169]. In this regard, the direct saponification and extraction considerably reduce solvent waste, while the Smedes method used non-chlorinated solvents (is a greener approach). Better recoveries for vitamin E are obtained when the analysis is performed before saponification step (e.g., modified Folch, Smedes). The authors were able to analyze α-tocopherol, cholesterol, and fatty acids all from the same extract and applied the optimized method to octopus, squid, mackerel, and sardine successfully. From the assayed samples, squid and sardine showed higher values of cholesterol and vitamin E, respectively. Interestingly, normal phase chromatography was used to assess vitamin E [169]. Saldanha and Bragagnolo also used normal phase chromatography. The authors used very mild conditions during saponification, which are paramount to avoid cholesterol oxidation. Also, they monitored cholesterol contents after heat treatment and demonstrated that it decreased significantly, with a simultaneous increase of the cholesterol oxides contents (i.e., 19-hydroxycholesterol, 24(*S*)-hydroxycholesterol, 22(*S*)-hydroxycholesterol, 25-hydroxycholesterol, 25(*R*)-hydroxycholesterol, and 7-ketocholesterol) [170]. Bauer and coworkers analyzed cholesterol and cholesterol oxides in milk samples using reversed-phase chromatography. [171]. The presence of cholesterol oxides can indicate the source and nature of the food, as well as the storage and processing conditions suffered by a commodity. The authors conclude that milk has physicochemical characteristics that make it more resistant to oxidation of cholesterol compared to other products of animal origin. In this regard, several sample preparation methods for cholesterol oxides have been detailed elsewhere [173]. Daneshfar and coworkers used dispersive liquid–liquid microextraction as an alternative to the extraction and clean-up steps in sample preparation [172]. In this case, ethanol was used as a disperser solvent and carbon tetrachloride as an extraction solvent [172]. This work is a fine example of parameter optimization during method validation; different dispersion (i.e., EtOH, acetone, and ACN) and extraction (i.e., CS_2_, CH_2_Cl_2_, CHCl_3_, and CCl_4_) solvents were tested, as well as variables such as pH, volume and time. However, the authors fail to explain how they obtain total cholesterol from a complex matrix (for example, a method must be able to free cholesterol from its esterified form) when no hydrolysis is performed (i.e., ensuring not just the mere quantification of unbound/free cholesterol).

It should be pointed out that though the chlorinated solvents are used in very small quantities, they are still classified by the IARC (International Agency for Research on Cancer) as possible human carcinogens (group 2B). Finally, Robinet and coworkers used a cholesterol esterase in an unrelated matrix to avoid chemical saponification [174]. In this regard, cholesterol esterases (most active at pH 7.0, 37 °C, and in the presence of taurocholate) and lipases (most active at pH 7.7, and 37 °C [175]) are commercially available.

#### Method Application Experience

We suggest two major points: i. that it is recommendable to perform the saponification first and then the solvent-aided extraction ii. a response surface design may be useful to optimize the length of the saponification treatment.

## 3. Determinations Designed for Feed and Feed Ingredients

### 3.1. Mycotoxins

#### 3.1.1. Recent Approaches for the Determination of Mycoxotins in Feeds

Mycotoxins are secondary metabolites mainly by fungi *Aspergillus, Penicillium, Fusarium* and *Alternaria* species, in stress situations, which involve changes in temperature, moisture or pH in plants [58,176,177]. Currently there are more than 400 types of mycotoxins as ubiquitous contaminants in a wide variety of foods [178,179], such as, corn, cocoa, sorghum, wheat, oats, rye, cotton, peanuts, coffee, dairy products, eggs, among others [180]. Among the best known are ochratoxin (OTA), zearalenone (ZEA), trichothecenes, aflatoxin B_1_ (AFB_1_), fumonisin B_1_ (FB_1_) and their metabolites. The last two are listed as carcinogenic by the IARC [181]. Mycotoxins, in general, are teratogenic, mutagenic, carcinogenic, and can possess an immunosuppressive effect in both animals and humans [178,182], which can be aggravated by factors such as the animal species, the concentration of the toxin and synergism existing among them, in addition to the health and nutritional status of the animal [182,183]. Also, the direct effects on health, including decreased weight gain, feed conversion inefficiency, reduced production, and a decrease of the food system profitability, the increase in feedstuff costs, medical treatments, and ineffectiveness when exploiting the genetic potential of animals [183].

At an organ level, in the liver, AFB_1_ can generate several metabolites, which include aflatoxin M_1_ (AFM_1_), which is transferred to milk, a complete food nutritionally, and which is vital in the development of the first years of life [184,185]. Also, the AFM_1_ is a compound declared as a carcinogen that is very resistant to pasteurization and freezing [180,183]. Therefore, being trawl compounds in the trophic chain, which involve the adverse effects on livestock production, with an obvious risk to the health of consumers, it stresses the need for laboratories to possess the ability to analyze a large number of analytes in a single sample. In this way, the amount of information can be increased, and a wider diagnosis can be made about the safety of the food and feed industry.

**Figure 4:**
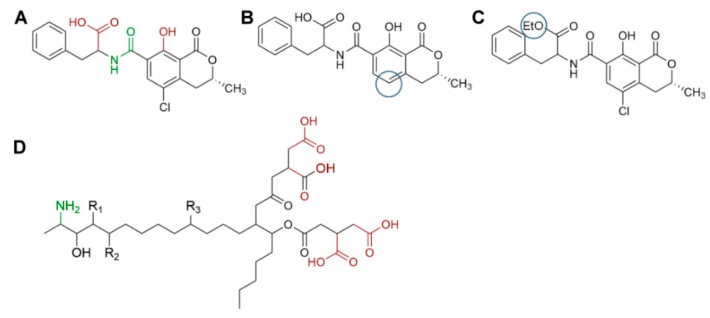
Chemical structures for (**A**) ochratoxin A, (**B**) ochratoxin B, (**C**) ochratoxin C, blue colored circles represent changes in the structure between ochratoxins, loss of Cl and OH in ochratoxin B and C respectively render a more lipophilic molecule. Et = C_2_H_5_, and (**D**) are the general backbone of Fumonisins. FB_1_ = 721.83 g mol^−1^ R_1_: H R_2_: OH R_3_: OH; FB_2_ = 705.84 g mol^−1^ R_1_: OH R_2_: H R_3_: OH; FB_3_ = 705.84 g mol^−1^ R_1_: H R_2_: H R_3_: OH; FB_4_ = 689.84 g mol^−1^ R_1_: H R_2_: H R_3_: H. Functional groups colored in green and red represent a positively and negatively ionizable moiety, respectively.

In this regard, Table 8 shows a summary of methods developed for the identification and quantification of mycotoxins, by different research groups, focused mainly on animal feed. For example, Njumbe Ediage and coworkers developed a technique capable of determining 25 mycotoxins in cassava meal, peanut cakes, cornmeal, and different sorghum varieties. The most exciting thing, in this case, is how the researchers solved the affinity fact of fumonisin and ochratoxin with the amino groups (due to the presence of carboxylic acid moiety, Figure 4) [177,186]. The researcher divided their extract into two portions, one to which formic acid and dichloromethane were added. After cleanup, the two independent shares were remixed evaporated at 40 °C, reconstituted with MeOH/H_2_O/CH_3_COOH, and 5 mmol L^−1^ CH_3_COO^−^ NH_4_^+^. During MS-based mycotoxin separations, flows are usually kept low, so solvent nebulization and evaporation are performed swiftly. The mobile phase is generally accompanied by an acetic or formic acid buffer to improve ionization especially for those compounds without readily ionizable functional groups (e.g., aflatoxins). Also, the formate ion is added in both solvents as one solvent depletes during the gradient separation and the buffer must always be present in a similar proportion [177,186]. Dzuman and coworkers and Rasmussen and coworkers, used, as an extraction method, a modification of the QuEChERS method, (Quick, Easy, Cheap, Effective Rugged, and Safe usually used for pesticide analysis). Both research groups coincide that QuEChERS adaptations for mycotoxin analysis open the possibility toward the simultaneous assay of several and distinct groups of contaminants (e.g., pesticides and mycotoxins) [179,187].

#### 3.1.2. Agricultural by-Products as Feed Ingredients

Agricultural and food-industry residues are valuable to animal nutrition as they are rich in many bioactive and nutraceutical compounds, such as polyphenolics, carotenoids and dietary fiber among others [190]. Agro-byproducts, used in animal feed, originate from perishable crops and, as such, are susceptible to fungal infection [191]. Hence, mycotoxin surveillance of these materials contemplating the most common contaminants present in such matrices, but also considering emerging contaminants (e.g., beauvericin, enniantins, and fusaproliferin) [191,192] is paramount. The food industry generally includes practices that guarantee the safety of the product meant for human consumption. Residues destined for animal production may not be subject to the same scrutiny. For example, the wine industry with a production estimated at 27 million liters worldwide. Presence of OTA in wine has been widely investigated [193]. However, with the development of new methods, it has been possible to find up to 36 different mycotoxins. (See for example, [194]).

Countries where the production of wine is the predominant, compared to other types of industry, a considerable amount of waste must be repurposed. As such, this might be of use as a ruminant (such as cows and goats) feed ingredient, where the pulp, husks, and seeds, might offer to the animal diet: fiber, energy, fatty acids, and antioxidant compounds which improve ruminal health, echoing in the quality of meat and milk [195,196,197]. As yet another benefit from this waste processing, the use of grape seeds as mycotoxin adsorbents has been investigated both *in vitro* [198] and *in vivo* (e.g., pigs [199]).

### 3.2. Antibiotics

#### 3.2.1. Recent Multiresidue and Multi-Class Analysis of Antibiotics in Feeds

Antibiotics are bioactive substances used against bacteria as a therapeutic, metaphylaxis or prophylactic agent both in humans and animals [200,201,202]. In livestock, some antibiotics are included in animal diets as growth promoter (e.g., monensin, narasin, ractopamine), decrease feed conversion, improve feed efficiency, and overall cost-effectiveness of animal production systems [203,204]. Overuse of veterinary pharmaceuticals in livestock, aquaculture, and the feed industry is reflected in the incidence of residues found in animal-derived food products (e.g., meat, eggs, milk, and honey) [201,205,206,207]. Antibiotic biotransference through the food chain may contribute to allergic reactions, mutanogenic and cancerogenic effects, found in humans and animals; additional to the growing rates of antimicrobial resistance [208,209]. Considering these issues, organizations worldwide (e.g., European Commission, United States Food and Drug Administration, World Health Organization) have generated protocols that help control, regulate and surveil the use of antibiotics in food-producing animals [208,210,211,212]. Hence, similar to mycotoxins, development of analytic methods that allow for identifying and quantitating a broad spectrum of compounds from a sample, directly contributes to surveillance programs for feedstuff manufacturing (raw materials or feed ingredients, compound feed, and premixes) and, similarly, those commodities derived from food-producing animals.

Table 9 shows a summary of the different characteristics of validated methods for the identification and quantification of veterinary antibiotics in different types of matrices. Molognoni and coworkers, optimized a method for the determination of spectinomycin, halquinol y zilpaterol in compound feed demonstrating once again the capabilities of mass spectrometry to assess two or more families of seemingly unrelated compounds. The authors tried both hydrophilic interaction and reverse-phase chromatography. Though HILIC (Hydrophilic Interaction Liquid Chromatography) offered good results, it requires a longer analysis time (i.e., up to 5 additional min), and is pH sensitive. Reverse-phase chromatography requires a relatively inexpensive column that is usually available in laboratories and which analytical instrumentation providers generally keep in stock. Additionally, a more effective separation was archived using heptafluorobutyric acid in the mobile phase [202].

#### 3.2.2. Multiresidue Analysis of Antibiotics in Foods

Barreto and coworkers developed a method to assay *n* = 14 different coccidiostats (i.e., lasalocid A, maduramicin, monensin, narasin, salinomycin, semduramicin, robenidine, diclazuril, toltrazuril, trimethoprim, chlopidol, amprolium, diaveridin y nicarbazin) in poultry muscle tissue and eggs; after testing several chromatographic columns, they selected the one that completed the separation under less time (i.e., 14 min). The authors used low temperature clean-up as an alternative to SPE, reducing costs, time and ion suppression. Internal standards where used to compensate intense matrix effects [207]. Regarding aquaculture, Kang and coworkers analyzed *n* = 41 antibiotics in fish muscle [205]. Similarly, Kumar Saxena and coworkers developed and validated *n* = 24 antibiotics (including quinolones, sulfonamides, and tetracyclines) in shrimp, and they preferred to use methanolic separation [206]. Finally, Shendy and coworkers identified *n* = 6 different classes of antibiotics in honey with a modified QuEChERs procedure simultaneously. Extraction was performed using ACN and MgSO_4_ and NaCl [214].

For both mycotoxins and antibiotics, a review was made of the wide variety of methods used in the food industry for the simultaneous, extraction of multiple analytes. For the identification and quantification of each chemical, a sensitive and selective tool is required. It is here that mass spectrometry has been useful, by reducing costs and response time. [185,202,209].

#### 3.2.3. Method Application Experience (Mycotoxins and Antibiotics)

A multitoxin (*n* = 26) analysis was applied to feedingstuffs using, as a reference, a method previously described by Wang and coworkers in cornmeal. ACN/CH_3_COOH/H_2_O (74:1:25) was used for extraction and cleanup we exploited the versatility of HLB cartridges (which allow the retention of a wide array of analytes with the least of interferences) [215]. When compared with immunoaffinity columns, this sorbent is less prone to fracturing and do not require low temperatures for storage. Later, the recovered extract was evaporated to dryness using vacuum at 60 °C and reconstituted with MeOH. The method relies on the 12.5-fold concentration of the original analyte to improve sensitivity. In the case of antibiotics (*n* = 23), we based our procedure on that described by Duelge and coworkers [216]. We extracted and eluted analytes using an ACN/MeOH solution. Again, we trusted the versatility of HLB SPE cartridges during cleanup. Both assays were single quadrupole equipped LC system using ESI^+^ and relied on a reverse phase separation (Zorbax Eclipse Plus, 100 × 3 mm, 3.5 μm). For mycotoxin separation, the mobile phase consisted of a gradient using acidified (0.1 mL/100 mL formic acid) ACN and H_2_O. For antibiotics, the gradient consisted of three different acidifed solvents ACN, H_2_O, and MeOH. In our experience, the first two-solvent gradient (starting with water) can separate most antibiotic families (β-lactams, tetracyclines, macrolides, streptogramins, lincosamides, aminoglycosides). Our gradient finishes with MeOH which is the only solvent capable of eluting coccidiostats (e.g., monensin and narasin). Efficient chromatographic separation was achieved under 35 min.

### 3.3. Amino Acids

Protein building blocks (i.e., amino acids), biologically, can be separated into two main groups. Exogenic/essential amino acids (i.e., Arg, Phe, His, Ile, Leu, Lys, Met, Thr, Trp, and Val), are not synthesized by the organism and must be provided in the diet to cover the requirement. The remaining amino acids are endogenic (i.e., Ala, Cys, Asp, Glu, Pro, Ser, Tyr, and Gly). Several of these amino acids (e.g., Lys, Met, Thr, and Trp) are prepared synthetically and are commercially available to use as feed additives. The purity of these additives must be routinely checked and adequately verified. Hence, methodological development is paramount for quality control for determination of amino acids in feed materials and feed mixtures. However, few reports have focused on feed. As a result; we intend to give an overview of the methods available in related matrixes.

#### 3.3.1. Fish Tissue

In a comprehensive research article, Mohanty and coworkers reported the complete amino acid profile (except tryptophan which was assessed spectrophotometrically and basic hydrolysis) for 27 different food fishes. [217]. Derivatization was performed using 6-aminoquinolyl-*N*-hydroxysucciminidyl carbamate (AQC), this specific reagent requires neutral pH to work. Adduct formation has the advantage of being stable and reacting with secondary amines. No variability among profiles was found in fishes of the same species from different locations. They also related the concentration of the amino acid found in the fish with the environment in which they live (e.g., marine and cold-water fishes showed relatively higher amounts of Met). At the same time, they recommend the consumption of certain fish species for several amino acids dietary deficiency in humans [217]. Example of methods suitable to analyze amino acids in diverse matrixes is shown in Table 10.

#### 3.3.2. Filamentous Cyanobacteria, *Spirulina* sp.

*Spirulina* sp. is a filamentous cyanobacterium that have been recognized for its nutritional value as a feed ingredient and supplement, and has been related to health benefits in humans [230]. Its nutrient profile has been reported previously, and it has even exhibited a higher amino acid value (except for Lys, Glu, Pro, His) when compared with that of soybean meal (a staple feed ingredient) [218]. Additionally, based on this profile, they calculated energy for a broiler diet. Nurcahya Dewi and coworkers applied different physical treatments (i.e., drying, sonication 30/60 min, reflux 60/90 °C, maceration in MeOH) to determine their effects on *Spirulina* sp. amino acid profile, which they concluded is rich in amino acids related to umami flavor (i.e., Asp and Glu). Drying and methanol maceration showed to be the treatment that delivered the highest (8.37 g/100 g) and lowest (2.34 g/100 g) contents of Glu, respectively [231].

Campanella and coworkers assayed total and free amino acids from *Spirulina* sp.; they found that freshwater *Spirulina* contained relatively high concentrations of non-essential amino acids. The authors indicate that the samples tested were lysine-rich and limited in sulfur-containing amino acids. Free amino acids constitute as high as 2% of the amino acid input. Method-wise, the authors used an oxidation-capable acid, this is chancy as it may contribute to analytes deterioration. Additionally, the mobile phase already included the derivatization agent [219].

Al-Dhabi and Arasu quantified polyunsaturated fatty acids, sugars, polyphenol and total and free amino acids in *Spirulina* sp. In contrast to the authors mentioned above, this research group used pre-column derivatization and a dedicated column for analysis. Total amino acids contents ranged from 11.49 to 56.14 mg/100 g; from which essential amino acids accounted for 17.00 to 39.18%. [220].

#### 3.3.3. Compound Feedstuff

For the specific case of feed, a time-reduced (i.e., complete separation in an eight-minute chromatographic run) analysis has been recently developed [225]. AOAC OMA^SM^ includes two different assays to determine amino acids based on LC; 992.12 design for pet foods using fluorescence and 999.13 include ninhydrin/Orto-phthalaldehyde (OPA) fluorescence or pulsed coulometric detection. Finally, a report made by Wang and coworkers described a successful simultaneous analysis of 20 amino acids without using derivatization using an evaporative light scattering detector [232]. More recently, underivatized amino acids have also been monitored using hydrophilic interaction liquid chromatography coupled with tandem mass spectrometry [233]. Herein, we included some examples of derivatization agents. However, we suggest the reader access a paper written by Masuda and Dohmae, which not only cites the four most commonly used reagents for amino acid derivatization, but also identifies their strengths and weaknesses [234].

#### 3.3.4. Bacterial Cell Walls, Peptidoglycan, and Food-Extracted Peptides

A less common application for LC, is to monitor the products from the hydrolysis of bacterial cell walls (using enzymatic physical, and chemical approaches) and posterior fragment analysis. Desmarais and coworkers design a method that included the digestion of Braun’s lipoprotein. Muropeptide fragments (monomers-trimers), 3,3-diaminopimelic acid among others [226]. Kühner and coworkers developed a similar application; complete muropeptide hydrolysis was accomplished within 24 h. UPLC/MS was used to monitor fragments. After BH_4_^−^ reduction, both Gram-positive to Gram-negative bacteria can be evaluated after gradient modification [227]. In this regard, MSD (Mass Spectrometry Detectors) serve as a good reference for additional mass information, which will ease the peptidoglycan *in silico* reconstitution. This application has not found accommodation in food or feed, but it can correctly be adapted for bioreactions/fermentations or lactic bacteria.

Other applications for LC include, for example, the work by Marseglia and coworkers. They identified *n* = 44 different peptides from cocoa beans. The peptide fragmentation pattern in fermented cocoa samples was used to describe the geographic origin, different fermentation levels, and roasting. Vicilin, a storage protein, was identified in cocoa bean samples, information that can be useful to understand the biological activity of cocoa and to determine the aroma relevant peptides [228]. MS assisted analysis is advantageous as amino acids lack any distinctive chromophores and already have readily ionizable moieties. Prados and coworkers recently have described a method to isolate, characterize and identify peptides that can downregulate adipogenesis. The authors also used semipreparative fractionation to achieve the initial peptide separation [229].

#### 3.3.5. Method Application Experience

When facing fresh feed products (e.g., wet pet food, forages) additional operation units such as lyophilization is necessary before sample treatment (see, for example, [235]). To obtain individual amino acids, most applications require acid or alkaline hydrolysis. However, amino acids are extremely susceptible to oxygen during hydrolysis, to prevent quantitative losses, we recommend the sample hydrolysis steps suggested elsewhere for furosine [236]. Additionally, pyrogallol in 1 g/100 mL is also used as a radical receptor (i.e., a radical sink) to avoid amino acid degradation. Particularly, Trp, Thr, and Tyr are usually lost during acid hydrolysis, cysteine is oxidized to cysteic acid, and asparagine and glutamine (if present) will transform to their respective acids. Hydrolysis may be performed using a feed of known concentration in parallel as a reference [237].

From the sample preparation standpoint, we have applied a Supelco ENVI-Carb SPE cartridge for cleanup as hydrolysate retain undesired particulates. A translucent extract is obtained after SPE that will be suitable for both FLD (Fluorescence Detector) and UV-Vis (Ultraviolet-Visible) detection-based analysis. Also, cleaner chromatograms are obtained as interferences are significantly reduced. This cartridge will adsorb (including those responsible for coloration) a great range of molecules, while the (charged) amino acids will not be retained. Sodium azide is applied routinely for extract storage. However, best results are obtained when measurements are performed immediately after preparation steps.

We have used a method based on OPA pre-column derivatization adapted from an established method for biopharmaceuticals [238]. We also recommend the strict use of a C_18_ guard column to increase column lifespan. When applied to feed samples and feed ingredients, essential amino acids covered include Arg, His, Ile, Leu, Lys, Met, Phe Val, and non-essential Ala, Asp/Asn, Glx, Cys/CY2, Gly, and Ser for a total of 14 amino acids. OPA derivatization is only effective under alkaline conditions (usually performed using borate buffer pH 8–10). Therefore, the feed hydrolysate must be neutralized (to pH 7.0) before injection, as the buffer will not be able to compensate for the [H_3_O^+^] that results from the acid treatment. Furthermore, 9-fluorenylmethyl chloroformate (FMOC) must be included during derivatization (additional to OPA) to obtain proline and hydroxyproline amino acids (see, for example, [224]).

Method automatization (when an automatic sampler is available) can concede an advantage since the reaction occurs in situ within the needle. Automated precolumn derivatization is also useful for unstable adducts (e.g., OPA derivatives). A benefit of amino acid derivatization is that most adducts can be monitored using a UV/VWD (Ultraviolet/Variable Wavelength Detector) or DAD/PDA (Diode-Array Detection/Photodiode-Array Detection), so even if the fluorescence detector is not available, analysis can still be performed. Though, the fluorescent detector can filter interferences, begetting cleaner chromatographs. We have also used the same method to assess the purity of feed grade amino acids, and taurine. The technique can also be applied to energy drinks to evaluate taurine in as a very simple “dilute and shoot” method after sonication for sample degassing. Interestingly, ninhydrin and OPA can detect complementary analytes to methods based in ninhydrin (see, for example, [223]).

### 3.4. Triphenylmethane Dyes

Malachite green is a dye usually used in aquaculture as a fungicide and antiparasitic due to its low cost and effectiveness [239]. The widespread use of this substance is not without downsides, though, including residue accumulation in fish tissue and contamination of sediments and water bodies, which can affect non-target organisms downstream (see, for example, [240,241]).

Recent and improved methods have found acceptance to monitor these kinds of dyes in fish tissue. For example, Hidaya and coworkers already conducted a short review on techniques available for detection of malachite and leucomalachite green in the fish industry [242].

**Figure 5:**
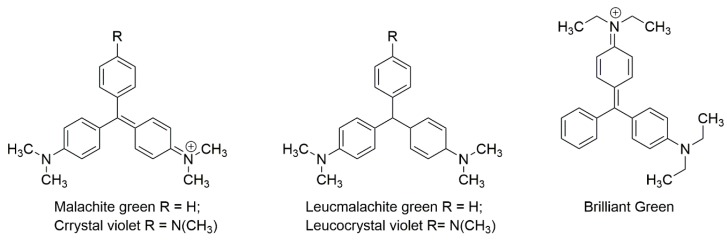
Chemical structures for three triphenylmethane dyes which are sharing a common phenyl backbone sharing a methylidyne. Each molecule has extended π-delocalized electrons justifying their crystal coloration and visible light absorption (ca. 621 nm for malachite green).

Within, this paper, several LC-based techniques are mentioned. Triphenylmethane dyes suffer from reversible redox reactions; each form can be oxidized or reduced to one another (see, for example, [243]; Figure 5).

Table 11 shows a summary of various methods for the extraction and identification of malachite green and its metabolites. Although it is a common contaminant in aquaculture production, and research focuses on fresh residues from aquaculture production animals (fish, shrimp, lobster, among others), the development of methods should also be extended to the analysis of feed [244] as fish and shrimp feed are made from marine by-products. Doses on fish or shrimp range from 0.05–0.2 mg L^−1^ as an active ingredient have been used. Treatments for fish eggs include dosages of 5 mg L^−1^ is usually suggested for fish tanks. Laboratories usually measure malachite green with equipment able to detect tissue residues below 2 µg kg^−1^ (maximum permitted residue limit in fish tissue; [250]). A very interesting approach was made by Furusawa who developed a green chemistry method for malachite green and its metabolite [251].

As previously mentioned, Wang and coworkers used solid-phase microextraction with the excellent result to assess malachite green, crystal violet and their respective metabolites using a monolithic fiber [245]. Bae Lee and coworkers homogenized fish tissue samples, and the extracted residues were partitioned into dichloromethane and an in situ oxidation with 2,3-dichloro-5,6-dicyano-1,4-benzoquinone. Afterward, cleaned-up was performed on neutral alumina and propyl sulfonic acid cation-exchange solid-phase extraction cartridges. Malachite green and crystal violet were determined at 618 and 588 nm using HPLC-Vis detector [246]. A common approach included analyzing dyes using traditional detectors and adding a step that included confirmation by MS. Chengyun and coworkers relied on Oasis^®^ MCX (a strong cation exchange-based adsorbent) to perform clean-up. After a two-step, QuEChERS extraction, dispersive solid phase extraction coupled with, both, a reverse phase and strong anion exchange (as well as a mixed mode adsorbent) cleanup was tested. Residues of the dyes were evaluated in codfish [247]. However, we do not see how anion exchange favors dye-stationary phase interaction, since all parent compounds are positively charged. Noteworthy, usually reverse phase columns can resolve these types of dyes with ease, even if several analytes are to be evaluated simultaneously. Croatia and Iran are specific examples of countries which have stated have found residues of this dye in fish tissue [252,253]. Both cases demonstrate the need to assess these compounds in food items. However, both research groups used immunoassays to evaluate the contaminant. AOAC method OMA^SM^ 2012.25 is a reference based on LC-MS/MS to assess triphenylmethane dyes and their metabolites in aquaculture products.

Additionally, US FDA reference method is based on the isolation of malachite green using alumina/propyl sulfonic solid phase extraction cartridges previous to Non-Discharge Atmospheric Pressure Chemical Ionization and an LC-MS^n^; quantification was performed in salmon [248]. Finally, since fish and shrimp compound feed can also be based in aquaculture by-product meal, as a source of protein, contaminated tissue can reach the final product. Hence, the need for feed analysis is evident, as it shows, Abro and coworkers [249].

## 4. The Common Ground among Measurements Performed in Food and Feed Laboratories

### 4.1. Nitrates and Nitrites

Nitrates and nitrites are natural compounds that are part of the nitrogen cycle, but especially high dosages of these ions are registered because of anthropological activities [254,255]; they enter human diets by means of drinking water, leafy vegetables, and cured meats. Noteworthy, these ions have been authorized as additives in several countries including the European Community [256,257].

Though there is evidence that both ions have a relevant biological and physiological function, special attention has been paid to nitrates and nitrites and their metabolites such as *N*-nitrosamines and nitrous oxide as all these molecules may pose a health hazard [256,257]. For example, these compounds have been related to colorectal cancer [256,257,258,259,260,261]. Hence, risk management and assessment in food have been proved necessary [258]. Regarding the quantification of NO_2_^−^ and NO_3_^−^ using HPLC, there are two main approaches used i.e., ion exchange and reverse phase columns (Table 12).

#### 4.1.1. Ion Exchange Chromatography

When analyzing crops, one must consider that cultivar, and harvest date can affect the nitrate levels of selected vegetables. Hence, maximum levels have been set by European legislation accordingly [262]. For example, Brkić and coworkers analyzed several leafy greens (*n* = 200) in two different seasons, in order to evaluate differences in ion content and encountered considerable differences among vegetable and sampling season [263]. Pardo-Marin and coworkers assessed vegetable-based baby foods, considering the levels found within these types of foods. They calculated ion ingestion between 13–18% of the acceptable daily intake for an infant. [264]. Quijano and coworkers assessed the nitrate content of vegetables (*n* = 533); they obtained values up to 3509 mg kg^−1^ in chard samples. They calculated an intake of 490 mg kg^−1^ bw day^−1^ for a young population, values which tend to increase the risk of exceeding acceptable intake values [265]. The main advantages in using ion exchange columns are that the separation can be accomplished using aqueous buffers which are made up from relatively cheap salts, making the methods apt for green chemistry and avoid mobile phase drift [263].

#### 4.1.2. Ion Pairing and Reverse Phase Chromatography

Tetrabutylammonium salt has also been used as an ion-pairing agent coupled with reverse phase columns (Table 12). For example, Hsu and coworkers used a reverse phase approach to assess both ions in cured meats and vegetables. The authors found the highest values of NO_3_^−^ in spinach (4849.6 mg kg^−1^) and for NO_2_^−^ in hot dogs (78.6 mg kg^−1^) [266]. Nitrite tends to oxidate to NO_3_^−^, the authors cite several factors affecting nitrate and nitrite recovery in foods (e.g., temperature, pH, metals). Usually, non-desired compounds found in greens differ from those found in meat products, for which proteins interfere significantly. Meat sample extracts will need pH adjustments and higher temperatures are needed to improve recovery. Some of these parameters must be monitored during analysis, especially when vegetables are subject of study [266]. Croituru used a similar approach to assess human, rabbit, rat urine as well as vegetables. However, they produced adducts (an azo dye, HO_3_SC_6_H_4_–N=N–C_10_H_6_NH_2_) based on Greiss reaction (sulfanilic acid form a diazonium cation (HO_3_SC_6_H_4_–N≡N^+^) with NO_2_^−^ and then with 1-naphthylamine) for NO_2_^−^ that was measured at 520 nm [267,268]. Interestingly, the authors followed the reaction with mass spectrometry. We encourage the reader to pay special attention to this paper as highlights difficulties during method development. The author concluded that while useful, the use of Greiss reaction, spectrophotometrically, is unadvisable as several samples may exhibit additional confounding compounds that may behave similarly as the NO_2_^−^ ion adduct. However, is quite valuable as a derivatizing agent when coupled with HPLC; the method can work with samples of different origins without the need for further modifications [267]. Samples were decolorized with carbon and ZnSO_4_ was applied for protein precipitation to overcoming this matrix interference and enhance the sensitivity. Croituru and coworkers used a validated method to assess NO_2_^−^ and NO_3_^−^ in vegetables for self-consumption; toxicologically speaking, the NO_2_^−^ content found in the samples was deemed too low to represent a hazard [269].

Stationary phases containing only alkyl chains have been used, but it is also possible to find mixed stationary phases, for example, Abdulkair and coworkers assayed NO_2_^−^ and NO_3_^−^ using a stationary phase containing both alkyl groups and phenyl groups (Table 12) to separate both ions successfully after sonication [270].

Chou and coworkers assessed both ions in vegetables and observed a high concentration variability was observed which reflect differences in environmental conditions [271]. The authors also optimized critical chromatographic parameters such as pH, organic solvent fraction, and flow [271]. In this regard, the methanol fraction optimization was demonstrated to be paramount to improve octylammonium solubility and achieve an optimal resolution between both ions. In contrast, pH and flow variations tend to have an effect only on chromatographic run times and not so much in resolution.

#### 4.1.3. Miscellaneous Methods for Nitrates and Nitrites

In contrast with ion pairing approaches, dos Santos and coworkers developed a method based on the reaction of the NO_2_^−^ with 2,3-diaminonaphthalene to yield a highly specific fluorescent 2,3-naphthotriazole adduct (λ_ex_ 375 λ_em_ 415 nm), under acidic conditions, to assess the ions in beetroot [274]. Cassanova and coworkers have developed an application for HPLC derivatization based on VCl_3_, 4-nitroaniline, methanesulfonic acid, and *N*-(1-naphthyl)-ethylenediamine. Under these conditions, a post-column reduction of nitrate to nitrite can be accomplished [275].

#### 4.1.4. Method Application Experience

**Figure 6:**
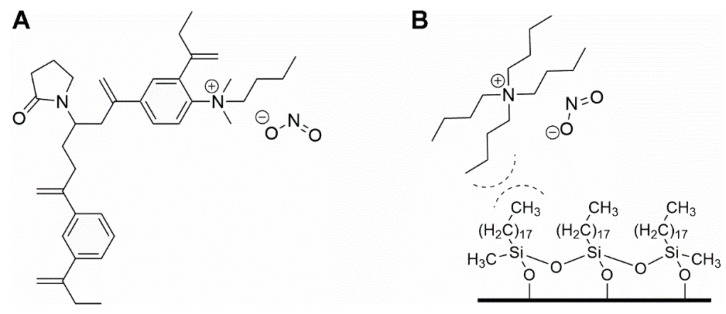
Schematic representation for the interaction of nitrite ion with (**A**) a cation exchange stationary phase or (**B**) interaction with TBAHS present in the mobile phase and stationary phase C_18_.

The preferred methodology used in our laboratories is based on the chromatographic determination of NO_2_^−^ and NO_3_^−^ anions simultaneously. Reverse phase (using a C_18_ column, i.e., Zorbax Eclipse 5.0 μm, 4.6 mm × 150 mm, set at 30 °C and 0.6 mL min^−1^) HPLC-PDA or -VWD (213 nm as the absorption spectra maximum) is sufficient to perform the assay [266,271]. It is important to emphasize that for the detection and separation of inorganic anions, in this case NO_3_^−^ and NO_2_^−^, the mobile phase must contain a complementary counter ion that interacts with it and with the bonded stationary phase of the column concurrently. In the absence of the counter ion, no interaction with the column is achieved and, as a result, no retention will be obtained at all, as the ions would come out in the void. In this scenario, a tetrabutylammonium salt (e.g., tetrabutylammonium hydrogen sulfate, TBAHS, 155837 Sigma-Aldrich) is a possibility (Figure 6B). In this case, the four alkyl chains from the reagent interact with the eighteen-carbon alkyl chains of the stationary phase and, at the same time, with the NO_2_^−^/NO_3_^−^. The elution order may be explained by considering a more delocalized negative charge (among three oxygen atoms) in NO_3_^−^ and the bent geometry of NO_2_^−^ due to the nitrogen atom-containing an electron lone pair. Interestingly, NO_2_^−^ is a larger anion (0.192 nm), when hydrated, than NO_3_^−^ (0.179 nm) [276]. Now, depending on the length of the column, the affinity of the this will not be sufficient to resolve peaks from the solvent front (specially the first peak; NO_2_^−^), this issue is easily solved including acetonitrile in the mobile phase, using slower flows, a longer column or even an ion pair agent with longer alkyl chains (e.g., octylamine). The mobile phase used is 20% acetonitrile, 80% TBAHS 5 mmol L^−1^, at a 6.5 pH. Interestingly, when injecting a solution with both ions present and at the same concentration, the response (the signals obtained), is very similar in area/height and, as such, sensitivity is very close for both anions.

**Figure 7:**
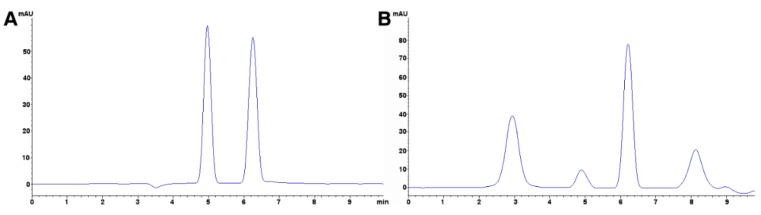
Chromatograph of (**A**) an aqueous 10 mg L^−1^ nitrite (4.95 min) and nitrate (6.26 min) standard (**B**) hay sample after extraction with hot water, SPE cleanup, and micropore filtration presence of nitrite (4.91 min) and nitrate (6.23 min) is evident.

The same methodology has been used in feed to assay hay samples (Figure 7A,B) that were presumed as the source of intoxication in horses [277]. In this case, from ten samples assayed, three (average concentrations of 92.77 ± 60.88 mg kg^−1^) and six (average concentrations of 92.13 ± 47.55 mg kg^−1^) samples tested positive for NO_2_^−^ and NO_3_^−^, respectively (unpublished data). Forage and swine compound feed samples (*n* = 10) have also been assayed with this method obtaining values from <5 to 23.69 and 2.30 to 4.96 and 925.15 to 1135.10 and 989.51 to 1479.71 mg kg^−1^ for NO_2_^−^ and NO_3_^−^, respectively on both accounts. In the case of feeds and fish meals, which suffer from severe matrix effects, SPE has been applied, with good results, as a cleanup and concentration step. Specifically, Oasis^®^ MAX cartridges, conditioned with 2 mL methanol, and 4 mL water, load 1 mL sample, wash 3 × 1 mL water, elute with 2 mL 0.5 mol L^−1^ NaCl solution. Chromatograms improve drastically when the elution from the cartridge is performed using the mobile phase.

#### 4.1.5. Legislation

Regulation 2002/EC/32 sets limits for NO_2_^−^ in fish meal (i.e., 60 mg NaNO_2_ kg^−1^) and complete feedingstuffs (i.e., 15 mg NaNO_2_ kg^−1^) excluding those intended for pets except birds and aquarium fish. We refer the reader to two thorough reviews that tackle regulatory as well as methodological topics [278,279].

### 4.2. Carotenoids

Chemically, carotenoids are conjugated hydrocarbons that may be further classified as carotenes (without any oxygen molecules) and xanthophylls (with one or more oxygen molecules). Carotenoids are widespread natural pigments, are recognizable from the bright colors (yellow, orange, red, or purple) that they often confer on plant and animal organ. The molecules responsible for producing said coloration must be attained from dietary sources. For example, lutein and zeaxanthin are carotenoid pigments that impart yellow or orange color to various common foods such as cantaloupe, pasta, corn, carrots, orange/yellow peppers, fish, salmon and eggs, β-carotenoid and isomer are found in sweet potatoes, dark leafy greens, butternut squash, lettuce, red bell peppers, apricots, broccoli, and peas, and lycopene are in tomato. As molecules with a conjugated double bond system, carotenoids serve several physiological functions (e.g., antioxidants, immunostimulants, photoprotection, visual tuning, among others). This electron delocation causes them to be particularly unstable compounds, especially sensitive to light, heat, oxygen, and acids. Hence, several precautions have been taken while extracting carotenoids. For example, must be carried out in dim lighting; use rotary evaporation at low temperature and reduced pressure also it has to be carried out under a stream of nitrogen. Finally, samples should be stored in the dark, at about −20 °C [280,281].

Carotenoids are fat soluble but, because of the high moisture content of plant tissues, a preliminary extraction solvent miscible with water (e.g., methanol or ethanol) is generally necessary to allow for penetration of the extraction solvent.

Saponification is required to remove interference as neutral fats, chlorophylls, and chlorophyll derivatives. Usually, this procedure is carried out with potassium hydroxide in methanol. Then, it is necessary to perform liquid–liquid extraction using a water-immiscible solvent (e.g., ethyl acetate, ethyl ether, hexane) to obtain the unsaponifiable fraction, where carotenoids should be present. [280,281,282,283,284,285,286,287,288,289,290,291,292,293,294,295,296,297,298]. The identification and quantification require high-resolution techniques; the reversed-phase high-performance liquid chromatography has been used routinely to determine carotenoids because of its satisfactory separation efficiency. So, several factors must be evaluated to employ HPLC technique such as column type, mobile phase, chromatographic conditions. Several methods for carotenoid analysis are summarized in Table 13.

Regarding column type, the analysis can be performed using a C_18_ column. However, YMC C_30_ Carotenoid dedicated column provides excellent results, had better resolution than a C_18_ column for separation of carotenoids and their geometric isomers. The thirty-carbon alkyl chains interaction with the carotenoid lipophilic profile guarantee less peak distortion and better resolution [280,281]. Compounds such as α/β/γ/δ/ε-carotene, lutein, zeaxanthin, β-cryptoxanthin, dehydrolutein, anhydrolutein, astaxanthin, galloxanthin, α-doradexanthin, adonirubin, and canthaxanthin can all be separated using the aforementioned chromatographic column.

According to Huck and coworkers, the flow rate did not significantly influence the resolution, but it is essential to use an adequate flow to generate acceptable column back pressure. Also, they studied the effect of column temperature on the separation of lutein, zeaxanthin, β-cryptoxanthin, and β-carotene. The column temperature was varied between 21 and 80 °C; the best selectivity being achieved at 21 °C, at a temperature of 34 °C, zeaxanthin could not be easily separated from lutein. The authors concluded that maintaining a constant temperature during carotenoid analysis is critical as small changes in the ambient temperature can cause significant changes in the chromatographic selectivity of the carotenoids and at temperatures higher than 60 °C, the investigated carotenoids unstable.

In the case of the mobile phase, the same authors indicated that carotenoid selectivity was better using tetrahydrofuran, rather than ethyl acetate, and also better than MeOH and ACN. Carotenoids are sensitive to degradation on the stationary phase of the column by the presence of silanol groups.

Solvent modifiers could be added to the mobile phase, for example triethylamine (TEA). Free silanol groups on the surface of silica deprotonate in the presence of the basic molecules, preventing the analyte from interacting with the medium. The TEA generates a positive impact on peak symmetry, reducing the peak tailing effect, reducing the retention time, and improving the recovery. The addition of triethylamine to the mobile phase can also have negative consequences, such as changes in the pH of the mobile phase; therefore, it is recommended that TEA be used in low concentration (less than 0.05 mL/100 mL) [299].

When using chlorinated solvents, the addition of ammonium acetate to the MeOH provides sufficient buffer capacity to prevent losses due to acid degradation of carotenoids. Some papers use MTBE as part of the mobile phase. The advantage in using this solvent, instead of chlorinated solvents, lies in the MTBE is less volatile (55.2 vs. 39.6 °C, respectively) and less toxigenic. Depending on the solvent system, a good compound separation may require a longer run time and poorer resolution compared with MeOH/ACN/H_2_O/CH_2_Cl_2_. Carotenoid content in tropical pigment-bearing fruits [281,295,300,301,302], and fish [302] have also been described.

#### Method Application Experience

The preferred methodology used in our laboratories is based on the work by Gayosso and coworkers with some modifications [282]. We use MTBE/MeOH as the mobile phase with a gradient system for 45 min with YMC C_30_ (150 × 3.0 mm, 3 μm) at 0.6 mL min^−1^ and 30 °C. These conditions were applied to identify and quantify carotenoids in food matrices such as palm oil, peach palm, sweet potatoes, papaya, and guava. We extracted the carotenoids from these matrices using a saponification procedure, followed by extraction with ethyl ether. This solvent evaporates at 40 °C and the residue is reconstituted in CHCl_3_. Undesired coextractants (e.g., waxes, sterol and tocophenol esters) are usually better solubilized with this solvent than MTBE saving from additional filtration steps and within-system precipitation. Optimization of injection volumes and initial composition of the mobile phase can somewhat mitigate the effect that injecting in a different solvent [303]. Analogous to polyphenols, carotenoid extraction methods must contemplate ester hydrolysis or other treatments to ensure the quantitation of overall amounts of carotenoids. For example, it is common to find carotenoid esters in food matrices, and these adducts present several intrinsic difficulties during carotenoid determination [295]. However, mass spectrometry-based LC is a powerful tool able to discriminate both parent compounds and their esters [295]. Recently, Wen and coworkers identified *n* = 69 carotenoids esters in *Physalis alkekengi* L. and *P. pubescens* L. fruits [297]. Additionally, BHA and BHT are common organic-solvent-soluble antioxidants to preserve carotenoid integrity [298]. Finally, our laboratory has also assessed carotenoid content in plasma from colored tropical frogs (*Agalychnis callidryas*).

### 4.3. Carbohydrates and Sugars Soluble in Ethanol

Animal feeds are, by definition, based on vegetable/plant sources that use carbohydrates as storage compounds, structure elements, and energy sources [10]. Then, carbohydrates form the most substantial portion of the organic matter in feeds; they can be divided into two main categories non-structural and structural carbohydrates. We encourage the reader to examine an excellent review of carbohydrate and organic acid in food commodities intended for human consumption by da Costa and Conte-Junior [304]. A great starting point for reviewing different approaches for carbohydrate analysis is the thesis written by de Goeij [305].

#### 4.3.1. Carbohydrate Measurement Using Amino-Based Columns

Xu and coworkers compared two methods for sample cleanup and extraction. A macroporous resin was compared to a solid phase sorbent based on alkyl chain. From the two approaches, SPE showed less analyte loss (11.32 vs. 0.69%). However, the discoloration ratio was similar for both methods. Sugar profile from molasses samples was obtained [305] after pigments, nitrogen compounds, and inorganic ions were removed. The analysis was performed using two NH_2_-based columns. Under the same conditions, it was concluded that the Zorbax Carbohydrate column showed better performance. Agius and coworkers recently developed a method to determine organic acid and sugars in tomato fruits [306]; the authors used ACN to improve peak shape. RID (Refractive Index Detector) is used for carbohydrate analysis since sugars do not have chromophores and alternative detectors (e.g., MS) are expensive. RID is the detector of choice in many labs for sugar profiling (Table 14) despite its relative lack of sensitivity. However, usual concentrations found in fruits counteract the issue.

#### 4.3.2. Carbohydrate Measurement Using Amide-Based Columns

Koh and coworkers developed a method using an amide-based column, which is designed to retain polar molecules [308]. Contrary to their amino counterparts, these columns can retain analytes wide range of mobile phase pH. Thirteen sugars were separated including monosaccharides, disaccharides, sugar alcohols. This separation is impressive since it includes several molecules commonly used as sugar substitutes or replacement sweeteners. Organic amines within the mobile phase are used as stationary phase modifiers [308]. The authors recommended the use of a 150 mm column as the reduction of time of analysis using shorter lengths, compromise resolution. However, peaks obtained on longer columns are typically wider peaks resulting in lower sensitivity due to increased diffusion.

#### 4.3.3. Carbohydrate Measurement Using Ligand Exchange-Based Columns

Duarte-Delgado and coworkers assayed four different extraction methods for sucrose, glucose, and fructose, and demonstrated that a double aqueous MeOH extraction was the more efficient approach for the determination of these sugars [310]. The authors used SPE and guaranteed the removal less polar compounds and avoid possible co-elution with sugars during HPLC analysis. Extraction method seems to be more critical for mono than disaccharides, and starch gelification appears to be an interference when extraction is performed with hot water. Zielinski and coworkers a cation exchange gel in calcium form column to determine sucrose, D-glucose, fructose, and sorbitol in different ripe stages and during senescence of *Malus domestica* (Suckow) Borkh [309].

Senescent apple juice showed higher sugar concentration; a stage in which fruit is better suited for fermentation. Shindo and coworkers used recovered sugars from samples such as orange juice, yogurt, chewing gum, milk, and biscuits (this last matrix needed a triple extraction to obtain adequate recoveries). Additionally, the authors optimized column temperature and flow rate [311].

#### 4.3.4. Reverse Phase Columns and Sugar Derivatization Techniques

Several detection systems are used to detect carbohydrate after chromatographic separation, an approach commonly used is the pulsed electrochemical detection. A thorough review of this technique has been already written by Corradini and coworkers [321]. Evaporative light scattering detector has also been used to assay sugars. Dvořáčkova and coworkers wrote a comprehensive review of this technique [322]. The most common detector for chromatographic analysis of sugars is refractive index. UV detection is usually inconvenient as the wavelength 210 nm (low range of the UV) has the disadvantage of exhibiting interferences. An easy way to circumvent this to derivatize using pyrazolones (e.g., 1-phenyl-3-methyl-5-pyrazolone) to form Schiff bases with reducing sugars and monitor using 248 nm. This approach only works for reducing sugars. Hence, sucrose will not be detectable. Additionally, a C_18_ column (usually readily available) can be used to separate the adducts. Canesin and coworkers analyzed sorbitol from lateral buds of fruit trees (e.g., black mulberry, peach, avocado, and pear) as a way to monitor primary photosynthesis products [312]. In this case, traditional detection systems are not useful as levels of sorbitol are in the µg per mg.

Hung and coworkers were able to add a fluorophore to aldol sugars assisting in their detection and mass fragmentation [313]. Naphthylimidazole fluorescent derivatives were obtained successfully for sugars (only for reducing aldoses) extracted from beverages such as fruit juice, yogurt, coffee drink, milk tea, and flavored milk. Additionally, oligosaccharides from a *Solanaceae* were identified using the approach above and NMR as an additional confirmatory tool. Recently, special attention has been drawn toward added sugars in food commodities; sterner regulations have been set in different countries due to population health concerns such as obesity, diabetes, and heart disease [323]. Hung and coworkers also used their approach to assess added sugar in the food items tested [313]. Carbohydrates analysis in food should contemplate, systematically, added sugars during chemical determinations [324].

#### 4.3.5. Aqueous Normal Phase Chromatography for Sugars

Interestingly, Valliyodan and coworkers used an aqueous normal phase approach based on a hydrophilic polymeric gel) to assess sugars from soybean. The addition of just 20–30 mL/100 mL of acetone to acetonitrile, in the mobile phase, permitted the successful separation of galactose from glucose [314].

#### 4.3.6. Complex Carbohydrates and Conjugates

Hydrolysis of complex (mainly structural) carbohydrates has been used previously to assess them [325,326] by indirect determination of their basic units and building blocks. Several approaches can be used to achieve this [325]. However, HPLC can be an attractive one since it provides high specificity and selectivity. As hydrolysis usually produces considerable concentrations of the monomer, usually sensitivity is not an issue. For example, we have used endo-1,4-β-mannanase (EC 3.2.1.78) to break down and indirectly determine mannan, monitoring mannose. Similarly, hydrolysis can be used to assess the quality of commercial mannanase. Mannanase is commonly used as a feed ingredient to improve nutrient absorption [10]. Here, an enzyme of known activity (a standard, see for example E-BMANN from Megazyme) is directly compared to the commercial one (the feed additive); a galactomannan polysaccharide (like guar gum) can be used as the substrate.

Weiß and Alt described an exhaustive method to assay sugars in plant materials and feeds. Separation of the following was achieved: inulin, verbascose, stachyose, raffinose, cellobiose, sucrose, isomaltose, maltose, lactose, glucose, xylose, galactose, rhamnose, arabinose, fructose, mannose, ribose, and mannitol [315]. Flow rate, temperature, mobile phase composition, and injection volume were optimized. From the series of columns tested, the Nucleosil^®^ Sugar 682 Pb (Macherey-Nagel GmbH & Co. KG, Düren, Germany) was finally used at 85 °C, H_2_O at 0.4 mL min^−1^, and using 20 µL. Recent data show that inulin-rich diets can benefit gut microbiome, notwithstanding, routine inulin analysis in feeds is uncommon [327]. It was not until very recently that the minimal performance requirements were established for fructans analysis in feed, pet food, and their ingredients [328]. However, excess dietary fructans have demonstrated adverse health effects in equines [329]. AOAC^®^ (Rockville, Maryland, USA) Official Method^SM^ 997.08 is available to assess fructans in food products using ion exchange chromatography with pulsed amperometric detection. The method is based on two-step hydrolysis using amyloglucosidase (to remove starch) and inulinase. Measurement of simple sugars in different food-derived extract fractions is performed using glucoheptose as an internal standard. The same principle has been used to assess fructose derived from fructans in pet food [330]. Verspreet and coworkers analyzed fructan from wheat grains after acid hydrolysis. Mild conditions used during hydrolysis avoid the release from other naturally occurring saccharides in wheat that would otherwise interfere during the fructan estimation (e.g., raffinose oligosaccharides) [316]. Correia and coworkers have developed a method to analyze fructooligosaccharides [317]. These sugars are dietary and are used as food ingredients (incorporated as dietary fibers in commodities). The authors monitored sucrose pathway fermentation products from *Aspergillus aculeatus* as a potential source of fructooligosaccharides; fructose, glucose, sucrose, 1-kestose, nystose, and 1^F^-Fructofuranosylnystose were monitored.

We have used enzymatic hydrolysis to obtain glucose from starch molecules present in feed and feed ingredients. Total and resistant starch was measured in several matrices including (e.g., silages) [318]. Bai and coworkers analyzed mono- and oligosaccharides from Hakka rice [319] as a measure of quality for sugars such as isomaltotriose, isomaltose, panose, maltose, and glucose. Finally, the determination of bacterial exopolysaccharides has also been reported [320,331].

#### 4.3.7. Method Application Experience

Amine-based columns (e.g., Zorbax^®^ Carbohydrate (Agilent technologies, Santa Clara, USA), Ultisil^®^ XB-NH_2_ (Welch Materials, Inc, Texas, USA)) are successful in separating mono and disaccharides in foods especially those containing lactose (such as dairy products). However, this type of stationary phase suffers easily from poisoning as amine functional groups form covalent bonding with several compounds (e.g., Schiff bases). As the amine functional group is sensitive to pH changes, extracts must be adjusted to avoid changes in the chemical form of the stationary phase functional group as this may affect repeatability/reproducibility or even obliterate the column capacity for retention. Hence, the elimination of interferences is paramount. Additionally, when retention capacity is lost, it is possible to apply changes in the mobile phase composition and flow (e.g., to increase acetonitrile concentration and reduce flow).

A particular case is that of coffee samples. Amine-based columns especially suffer when analyzing coffee extracts as they contain phenolic acids (e.g., chlorogenic, syringic, ferulic, protocatechuic and hydroxybenzoic acid) and alkaloids (e.g., caffeine, caffeic acid, theophylline, trigonelline) [332]. Costa Rican regulations accept not more than 10 g/100 g sucrose in roasted coffee. Hence, monitoring sugar levels, as a quality standard, in these products is paramount. When routine quality control in coffee samples is necessary, we recommend to use stationary phases more resistant to pH changes (e.g., amide-based), include mobile phase modifiers (e.g., triethylamine), or intensive extract clean up.

**Figure 8:**
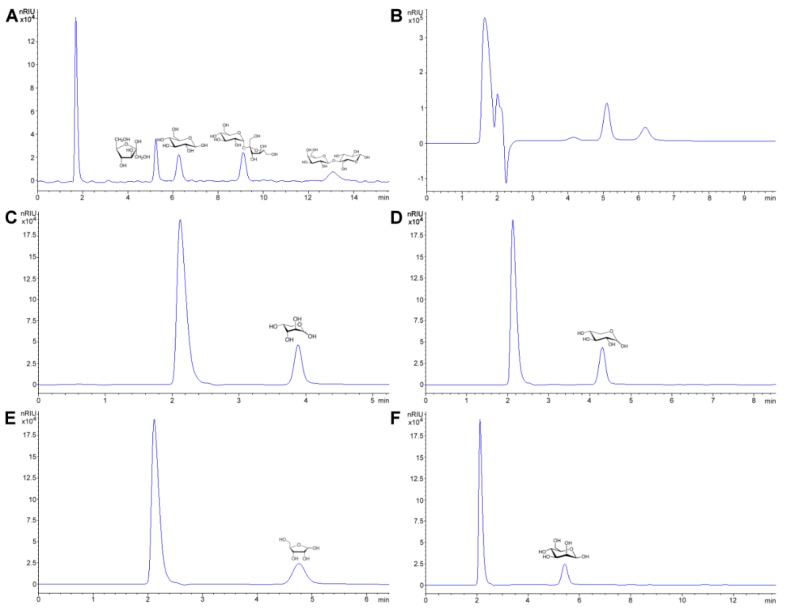
Chromatographs of (**A**) 2 g/100 mL standard mixture of four sugars including fructose (5.24 min), glucose (6.26 min), sucrose (9.12 min), and lactose (13.09 min) separated using amino column (Zorbax Carbohydrate, 0.7 mL min^−1^, 80 ACN: 20 H_2_O). (**B**) Sugar content of a molasses sample after hot water extraction, fructose (5.18 min) and glucose (6.31 min) signals are evident. (**C**) 1 g/100 mL standard solution for arabinose (3.89 min) (**D**) 1 g/100 mL standard solution for xylose (4.30 min) (**E**) 1 g/100 mL standard solution for ribose (4.76 min), and (**F**) 1 g/100 mL standard solution for mannose (5.42 min). Signal at ca. 1.80 min corresponds to the solvent front; constant in all injections.

**Figure 9:**
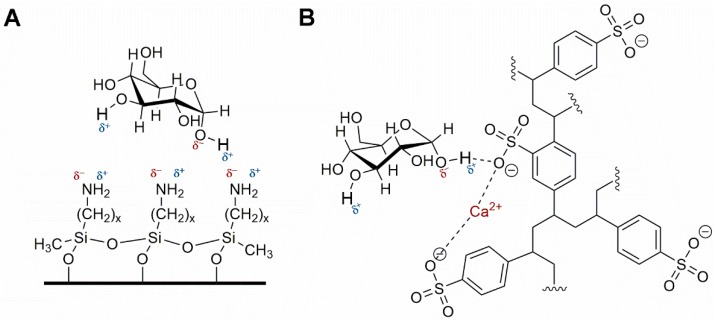
Schematic representation of sugar interaction mechanism using (**A**) amine based (**B**) calcium ion-based ligand exchange column.

In the case of animal compound feed, for example, suckling pigs feed usually contain lactose. Contrary to the amine-based column (Figure 8A,B and Figure 9A), ion exclusion (e.g., Agilent Hi-Plex Ca, Phenomenex^®^ Rezex^TM^ RCU-USP Ca^2+^ (Torrance, California, USA)) is better equipped to deal with a larger range of samples and is less prone to deteriorate.

These types of columns are able to separate H^+^ (organic acids/monosaccharides), Na^+^, Ca^2+^ (sugars/alcohols), Ag^+^ and Pb^2+^ (oligosaccharides). Sugars and alcohols are separated using ligand exchange (Figure 9B) and organic acids by ion exchange. During ligand exchange, the more complex sugars elute first whereas simple ones, such as fructose, elute last, opposite to the elution order found in amine-based columns. Another advantage is that ion exchange columns need ultra-high purity water (type I) to segregate analytes while amino-based columns require acetonitrile in the mobile phase to perform. However, amino-based columns have the inherent advantage that complete chromatographic runs can be achieved under 12 min (while setting the column at 30 °C). Meanwhile, a good separation using exchange columns can be extended up to 30 min at 60 °C. As LC-MS usually use low flow rates to aid in solvent nebulization, it is harder to develop methods using this type of column due to their dimensions. When using ion exchange based-columns, EDTA can be used to sequestrate ions present on complex sample extracts reducing interferences. Peak tailing or fronting is also common when there is already wear of the chromatographic column. Finally, mathematic designs can be used to optimize method critical parameters and attributes, at least one research group has used this approach (i.e., Monte-Carlo simulation) to analyze sugars in herbs [333].

### 4.4. Organic Acids

Organic acids are common substances found naturally in several foods and result from fermentation processes [334]. As such, they are responsible for the particular flavor and aroma of commercially relevant commodities such as wine, vinegar, fermented meats, and yogurt, to name just a few. These substances have found widespread use in the food and feed industry as preservatives (increasing shelf-life, [335]) and antimicrobials (due to their bacteriostatic properties) (large-scale use of benzoic acid in beverages is a clear example, [335]). Organic acids can be determined by HPLC using ion exchange columns. Several anion exchange columns have already been mentioned in the previous section. Usually, though, measurement is commonly made using a UV detector at 210 nm (detection of absorption of carboxyl groups). Solvents and columns may vary slightly, but an isocratic method is sufficient to separate the analytes. The sample preparation for organic acid determination in beverages can be straightforward. In some cases, depending on the clarification, process suffered by the final product, it may consist of centrifugation and microfiltration. For an excellent primer for organic acids, we recommend the book edited by Vargas [336].

#### 4.4.1. Reverse Phase Chromatography Analysis in Foods

Neffe-Skocińska and coworkers analyzed sugars, ethyl alcohol and organic acids in Kombucha tea beverages (a fermented brew) with an emphasis on glucuronic acid (which has been associated with health benefits) [337]. The fermentative profile was evaluated for 10 days at 3 different temperatures. Nour and coworkers use low temperature so they can separate 6 compounds (oxalic, tartaric, malic, lactic, citric, and ascorbic) in 13 min. The applied temperatures (i.e., 10 °C) in a 250 mm column using 0.7 mL min^−1^ flow rates ensures optimal resolution of the compounds while obtaining adequate peak shapes within a reasonable time. A buffer adjusted at 2.8 pH guarantees that the compounds of interest are maintained during chromatography as protonated species [338].

Different citrus juices were tested finding concentrations of citric acid ranging from 7.39 × 10^4^ to 6.89 × 10^4^ mg L^−1^. Reverse phase separation of acids uses buffers (e.g., the H_2_PO_4_^−^/HPO_4_^2−^ pair) or salts (e.g., Na_2_SO_4_) to accomplish separation. The advantage of this approach is that usually C_8_/C_18_ columns are readily available and are relatively inexpensive. The downside resides in that the use of this kind of mobile phases increase the possibility of crystal precipitation in the pump and capillaries. The buffer has to be prepared daily (to circumvent microbial growth), and pH values strictly supervised (to avoid retention time shifts). Lobo Roriz and coworkers determined organic acids in three different medicinal plants which are widely consumed as infusions. *Gomphrena globosa* L. showed the highest levels or organic acids (mainly malic and oxalic) [339].

*Pterospartum tridentatum* (L.) Willk. and *Cymbopogon citratus* (DC.) Stapf showed higher levels of citric and succinic acids, respectively. Acid content depends on inherent plant genetic characteristics and edaphoclimatic conditions. The authors also analyzed sugars (using HPLC-RID Eurospher 100–5 NH_2_ column and melezitose as internal standard) and, interestingly, tocopherols (α, γ, and δ-tocopherol, normal phase YMC Polyamide II column and fluorescence at λ_ex_ and λ_em_ 290 nm and 330 nm). Scherer and coworkers used a reverse phase column to assess ascorbic acid stability in apple, orange and lemon juices. They also compared nutritional analysis reported within the food labels for ascorbic acid with that obtained experimentally [340].

#### 4.4.2. Ion Exchange Chromatography Analysis in Foods

Llano and coworkers analyzed sugars, acids, and furfural in pulp mill residue. The authors used a resin-based cross-linked gel column for low molecular-weight chain acids, alcohols, and furfurals. This method is particularly interesting since a comparison between 2 sets of columns for each application was tested (Table 15). The authors also included specific details for each column and optimize temperature, injection volume, and flow rate. Size exclusion also seem to have a role in sugar separation using ion exchange columns [341]. Though this application is not specifically for food, xylooligosaccharides (from revalorization alternatives for materials derived from the pulp mill enterprise) have found applications in the food industry and have been even linked to health benefits [342]. Saleh Zaky and coworkers reported a simultaneous analysis of chlorides, sugars, and acids [343]. However, the paper states that several inorganic ions are retained with the same strength within the column (all tested ions have different physicochemical properties; i.e., hydration spheres, charge among others). We have not been able to repeat this procedure. The authors did analyze sugars and acids (i.e., citric, lactic, acetic) and ethanol in a grand variety of food products including energy drinks, sodas, tomato juice and sauce, brine, milk, whey, cheese, and hummus. An interesting paper focused on the determination of organic acids from olive fruits. Different organic acid profiles were found for unique fruit varieties. They found oxalic, malic, succinic, and citric as main organic acids [344].

Mihaljević and coworkers separated organic acids in wine. Organic acid profile (especially glucuronic and galacturonic acids levels) was able to distinguish among Traminer vs. Welsch produced Croatian wines. Mobile phase rate was reduced during chromatography when target acids were glucuronic, gluconic, galactaric, and galacturonic [346]. Diacids and citric acid considerably differ structurally (e.g., number of carbons). Meanwhile, the reduction of flow rate responds to the subtle differences among these intimately related structures, making them more difficult to resolve. Sánchez-Machado and coworkers preserved shrimp tissue through fermentation with lactic acid bacteria. A complete separation of lactic, citric and acetic acid was accomplished. Sonication time and initial sample mass were optimized during the assay [347]. Finally, though most of the tests regarding organic acids extraction-wise are straightforward, even in brightly colored samples (e.g., fruits [350]), still SPE cleanup has been applied, with adequate recoveries, to these extracts to remove interferences as anthocyanins and carbohydrates that may co-elute during acid analysis (especially relevant if a non-selective detector is used) [345].

#### 4.4.3. Ion Exclusion Chromatography Analysis in Foods

Fasciano and coworkers modified a reverse phase column, a C_18_ column was dynamically modified by running a solution of SDS through the column (Table 15). They separated organic acids after optimizing sulfuric acid concentration, flow rate, and pH; an example of how a reverse column can be made more versatile. A wide array of compounds in juices and sodas were analyzed using ion exclusion chromatography [349]. For a detailed description of ion exclusion chromatography, we encourage the reader to pay special attention to this paper introduction.

#### 4.4.4. Silages

The maturity of the crop governs silage quality at harvest. However, fermentation in the silo further influences the nutritive value of silage. Coblentz and Akins recently published a detailed discussion of silages [351]. Similarly, Khan and coworkers wrote a more specific review based on maize silages [352]. In both papers, references to silage quality based on organic acids are mentioned. Since silage is the result of this fermentation process, the organic acid analysis is used to monitor its quality. Concentrations of fermentation acids do not seem closely related to silage intake; however, they are decisive in the balance of volatile fatty acids produced in the rumen. In turn, affecting gluconeogenic metabolism and influencing milk and body composition in productive livestock. Several researchers have dedicated efforts to not only assess organic acid concentrations from silages but also have studied the effect that organic acid has on silage fermentation. For example, Ke and coworkers included malic or citric acid at concentrations of 0.1 to 0.5 g/100 g during alfalfa ensiling of alfalfa and concluded that these levels improved silage fermentation quality [348]. Additionally, both acids can be further used as feed additives that have proven to promote animal performance. Silva and coworkers determined the fermentation profile of alfalfa silages treated with microbial inoculants at different fermentation periods under tropical conditions [353]. From the strains tested *P. pentosaceus* showed the most efficiency suggesting its use as a silage inoculant. The sample pretreatment just consisted of extract acidification with metaphosphoric acid, gravity-aided filtration, and centrifugation.

#### 4.4.5. Method Application Experience

**Figure 10:**
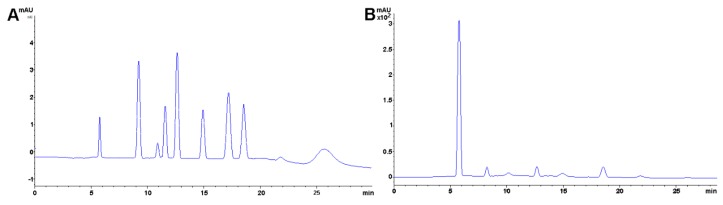
Chromatographs of (**A**) Mix of organic acid standards malic acid (9.24 min) methanoic acid (formic acid, 10.92 min), ethanoic acid (acetic acid, 11.65 min), propanoic acid (propionic acid, 12.62 min), lactic acid (14.92 min), 2-methylpropanoic acid (isobutyric acid, 17.22 min), butanoic acid (butyric acid, 18.52 min). (**B**) A silage sample after extraction with acid 0.01 mol L^−1^ H_2_SO_4_. Fermentation products identified at 18.499 min, 14.903 min, 12.606 min. The signal at ca. 5.70 min corresponds to the solvent front.

We have used ligand exchange-based analysis to routinely screen silage quality (Figure 10). Sample pretreatment consists of metaphosphoric acid extraction. We also have taken advantage of sample extraction for ammoniacal nitrogen (a modified version of method 941.04). In this type of columns, poly and diacids are eluted first. Monocarboxylic acids will elute later on during the chromatographic run in order of increasing alkyl chain length (i.e., formic, acetic, propionic, butyric). Finally, we have used liquid chromatography coupled with a variable wavelength detector set at 210 nm, a Hi-Plex H (300 × 7.7 mm and 8 µm particle size) column kept at 60 °C, and a 50 mmol L^−1^ H_2_SO_4_ solution with a flow rate of 0.6 mL min^−1^ to monitor ammonium propionate, added as a preservative, in dry dog foods (see for example, [354]). Average concentrations of (693.12 ± 75.63) mg kg^−1^ have been obtained for local products. Sieved (at 0.5 mm particle size) dog food was treated with hot water to extract the propionate quantitatively. Both RID and UV detectors can be used for both organic acid and sugar (and alcohol) analysis. Using RID will enable the user to monitor all the compounds above simultaneously, but RID detectors suffer from low sensitivity when compared to others.

### 4.5. Vitamins

Vitamins are essential micronutrients that humans and animals need for normal metabolism. The lack of these nutrients in dietary sources can cause serious disease; trace amounts of these compounds are required for growth and reproduction. Based on their solubility, vitamins have been divided into two groups: those soluble in organic non-polar solvents and water-soluble vitamins. Thus, the vitamins from the B-complex and vitamin C are classified water soluble while the fat-soluble vitamins are isoprenoid compounds, namely vitamins A, D, E and K. This last group is found in small amounts on foodstuffs, associated with lipids; stored in the liver and fatty tissues, and are eliminated slower than water-soluble vitamins [45].

#### 4.5.1. Fat-Soluble Vitamins

Vitamin A is commonly expressed as retinol equivalents but can occur in different chemical forms, i.e., retinal, retinoic acid and retinyl esters. In foods, it is very common to find this vitamin as retinyl esters, more specifically, as acetate, propionate, or palmitate [355]. This vitamin is involved in immune function, vision, reproduction, and cellular communication [356]. Vitamin E is a term used to designate some related compounds as tocopherols and tocotrienols, it is found in fat products of vegetal origin, mainly oils. The most common tocopherols that can be found in food and feed are α/β/δ/γ-tocopherol in different proportion. Mixed tocopherols are considered the most effective lipid-soluble antioxidants [357]. Vitamin D is naturally present in very few foods like sea products, eggs, meat, and dairy products, the most commonly found members are vitamin D_2_ and D_3_, but it is also produced endogenously when ultraviolet rays from sunlight strike the skin and trigger vitamin D synthesis from 7-dehydrocholesterol [358]. It promotes the absorption of calcium, regulates bone growth and plays a role in immune function [359]. Lastly, vitamin K is an essential nutrient for animals and humans because it is required for functioning of the blood clotting cascade [360], just as vitamin D, vitamin K can be found in two forms i.e., phylloquinone (vitamin K_1_, found in green plant leaves e.g., spinach, collards, lettuce, and broccoli [361]) and menaquinone (vitamin K_2_, bacterium residing in the vertebrate intestine [362]).

Since these compounds are involved in metabolic pathways, and are paramount in health promotion in animals and humans, it is crucial to determine their content in food and feed to comply with daily requirements and quality control. That is why several studies have been conducted regarding the extraction and quantitative analysis of these vitamins, either individually or simultaneously [359,363,364,365,366,367,368,369,370,371,372,373,374,375,376,377,378,379,380,381,382].

##### Sample Preparation

Most of the analytical methods involve previous steps of sample preparation like saponification, solid-liquid or liquid–liquid extractions, followed by a concentration step before HPLC analysis (Table 16). The sample pre-treatment is critical for an accurate method. That is why there are many aspects that need to be controlled, Qian and Sheng have studied seven different variables to take into account for simultaneous analysis of vitamins in animal feed. These variables were related to the extraction procedure: (1) sample particle size, (2) solvent, (3) the ratio of sample to solvent, (4) extraction with and without N_2_ protection, (5) extraction time, (6) equipment and (7) the use of SPE for cleanup [367]. They evaluated how each of the variables affected both the coefficient of variation and the recovery of each of the vitamins in order to obtain extraction conditions that would allow them to satisfy each of the vitamins in a satisfying way.

Regarding the first variable in meals and flours, subsample variability and homogeneity is closely linked to particle size. Qian and Sheng observed that large sample particle size causes an incomplete vitamin A extraction with high variability [367]. We have noted that for fresh products, with a total fat content greater than 10 g/100 g and high moisture content (i.e., greater than 85%) (e.g., avocado (*Persea americana* Mill.) and peach palm (*Bactris gasipaes* Kunth)), it is advisable to freeze dry the sample, before analysis, to promote homogenization and eliminate water that interferes with fat-soluble compound extraction.

Qian and Sheng developed the assay procedure without saponification, but nevertheless it is a widespread procedure used in the analysis of vitamins since it is an efficient way of removing interferences of lipid origin that can be found in the matrix [363,365,368,374,378,379,382]. Since it is based on an alkaline digestion (i.e., heated KOH or NaOH aqueous or alcoholic solutions) there is a disadvantage, the saponification could generate oxidation of the vitamins, which translate into a loss for vitamin degradation and low recovery percentage [366,368]. Some researchers have made use of the antioxidant ability of some compounds such as BHT, BHA, TBHQ, ascorbic acid or pyrogallol to reduce oxidation losses [363,365,367,368,371,374,379,380]. Nevertheless, saponification procedures take time, and the extractions procedures are not always straightforward, because emulsions are generated, as Lim and co-worker mentioned in their comparison of extraction methods for determining tocopherols in soybeans [369], these inconveniences introducing considerable variation, low recovery, and reproducibility, situations that we have also seen in the development of this type of methodologies.

Recently, alternatives to saponification process for the extraction of vitamins have been used, some papers use enzyme-catalyzed hydrolysis and alcoholysis of ester bonds in vitamin A and E esters to facilitate their determination in milk powder and infant formula. They assayed six lipase preparations and one esterase preparation using diisopropyl ether, hexanes/ethanol and supercritical CO_2_ containing ethanol. Three of the lipases’ preparations from *Candida antarctica* (Novozyme 435), *Rhizomucor miehei* (Lipozyme IM) and *Pseudomonas cepacia,* showed considerably higher activity toward retinyl palmitate but there was no observed activity with α-tocopheryl acetate [372]. In feed, Xue and co-workers applied enzymolysis instead of saponification with a basic proteinase named Savinase in 30 min of incubation time at 40 °C getting good results in the determination of four fat-soluble vitamins (K_3_, A, D_3_, E) [366].

With respect to the solvent type and ratio solvent: sample, there is a wide variety of solvents available for the fat-soluble extraction, most of the methods use solvents such as hexane, heptane, chloroform, dichloromethane, ethyl acetate, tetrahydrofuran, ethyl ether, and the choice will depend on the type of matrix to work with (Table 16). For example, in the case of animal feed [367], a poor resolution was observed using hexane and chloroform, generating an overestimation of vitamin D, such mixture does not allow a good separation during centrifugation which produced a high %RSD.

If a mixture of acetone/CHCl_3_ (30:70) is used, the results in terms of variability and recovery of vitamins are outstanding, mainly for vitamin A. In low-fat matrices (less than 0.1 g/100 g, e.g., fruit juices), this solvent system has the disadvantage of generating emulsions and, hence, low recoveries. In the case of dairy and infant formulas where the presence of milk proteins is a hindrance, the extraction of the lipid part has been reported using saponification and extraction with hexane, leading to vitamin degradation in fat. It is also an extensive process [373,374]. For this reason, a group of researchers developed a fat extraction methodology using a mixture of CH_2_Cl_2_:EtOH 2:1 and separation at 4 °C with a centrifuge, giving satisfactory results for analysis of FAMES so it could be applied in the extraction of vitamins in these matrices [375].

Regarding extraction time and equipment, Qian and Sheng used vortex mixer for several minutes, rotatory mixer and supersonic mixer, these last two methods were not as effective for extraction of vitamin A and other vitamins due to low recoveries [367]. Hung used a rotatory mixer for extraction of vitamins D_2_ and D_3_ during one hour [376]. We have found, that for foodstuffs, the most efficient sample treatment is to rely on the combination of a vortex mixer for one minute, a rotatory mixer for 30 min or supersonic mixer for 15 min.

Effective extraction can be aided if the solvent contains a percentage of an appropriate antioxidant. N_2_ has been used in some protocols to the protection [377,380], of extracted vitamins from degradation because the solvent vapor that replaces air over the surface of extraction mixture has a protective antioxidant effect [367]. Qian and Sheng showed evidence that this protection did not influence in the mean values of vitamins A, D, and E and pro-vitamin D, but decreased the variation coefficient [367].

##### Chromatographic Analysis

The analytical method for the determination of vitamins in food and feed, is liquid chromatography (HPLC or UPLC) due to its, selectivity, short time of analysis, and high resolution. Methods based on chromatography can be easily automated and can determine several compounds at the same time.

Methods range from using normal phase chromatography with silica columns to reverse phase chromatography with C_8_, C_18,_ and C_30_ columns (Table 16). Lee and coworkers studied three different columns to separate vitamin A and E: an NH_2_ column, C_30,_ and C_18._ Concerning the resolution, they observed the β-tocopherol and γ-tocopherol peaks of vitamin E were not separated and appeared as a single overlapping peak when using a C_18,_ but it could be separated using an NH_2_ column. Regarding detection and quantification limits the NH_2_ column presented values lower than C_8_ column but higher than C_18_.

The solvent systems to use as mobile phase vary depending on the selected approach, in the case of normal phase chromatography the solvents systems mostly used are 2-propanol/hexane in different proportion, but also can be use methanol/hexane/THF (97.25:2.5:0.25), or hexane/MTBE (96:4) [374].In reverse phase the most common are MeOH-H_2_O, MeOH-ACN, both techniques can be used in gradient o isocratic mode.

**Figure 11:**
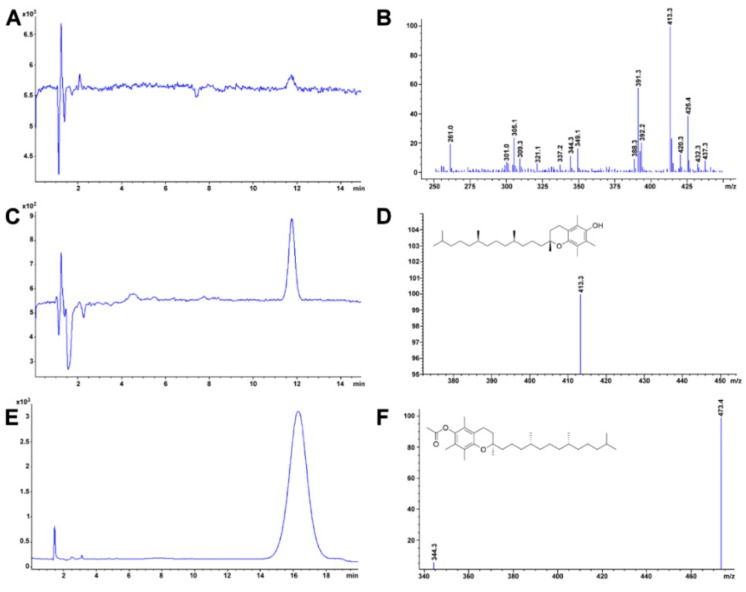
Single quadrupole LC/MS ESI^+^ chromatographs of (**A**) Total ion chromatogram α-tocopherol (a 1 mg L^−1^ solution in butanol) signal positively identified at 11.75 min (**B**) Mass spectra for α-tocopherol (a 1 mg L^−1^ solution in butanol) using a cone energy of 120 V extracted from a signal with a retention time of 11.71 min (**C**) α-tocopherol (retention time 11.77 min) identified in a chicken plasma sample after extraction with chloroform and butanol (**D**) α-tocopherol in selected ion monitoring (SIM) mode using a cone energy of 120 V extracted from signal with a retention time of 11.82 min (**E**). α-tocopherol acetate in an injectable vitamin E solution for veterinary use using a “dilute and shoot” approach (16.32 min), and (**F**) α-tocopherol acetate in SIM mode using a cone energy of 60 V extracted from signal with a retention time of 16.34 min.

As mentioned before, the liquid chromatography technique has a wide variety of monitoring techniques including PDA, FLD, ECD, ELSD or MSD. The most commonly used detector for vitamins is FLD, which is considerably more sensitive and selective than UV. Therefore, it is possible to carry out a simultaneous determination of vitamin A and E, for which a programming of the equipment is required so that at certain time intervals it uses the excitation wavelength (λ_ex_) and emission wavelength (λ_em_) specifies for each vitamin, for vitamin E, λ_ex_ = 285 and λ_em_ = 310 nm, for vitamin A the configuration at λ_ex_ = 325 and λ_em_ = 470 nm, but no other vitamins could be detected such as K or D. Alternatively, PDA can work with multiple UV wavelengths and determine the four vitamins at the same time. Mass spectrometry coupled chromatography is usually the most versatile option. However, it requires that the laboratory has the resources for its acquisition. We have successfully applied mass spectrometry to assess tocopherols in feed supplements and animal biological samples (Figure 11A–F).

#### 4.5.2. Hydrosoluble Vitamins

**Figure 12:**
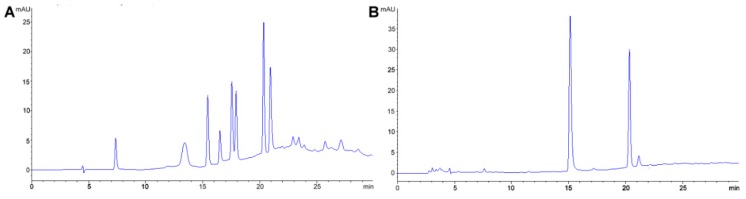
Hydrosoluble vitamin analysis based on ion pairing [383]. (**A**) Successful separation of 7 complex B vitamins including niacin (nicotinic acid, B_3_, 6.67 min), FMN (B_2_, 14.12 min), pyridoxal (B_6_, 17.007 min), pyridoxamine (B_6_, 18.607 min), pyridoxine (B6, 19.963 min), folic acid (B9, 20.630 min), and thiamine (B_1_, 25.074 min). (**B**) Analysis of a vitamin premix destined for feed formulation. Another advantage presented is that the separation can be performed using a reverse phase C_18_ column.

One of the main issues that the hydrosoluble vitamins analysis exhibit is that each molecule is structurally different. Hence, to assess each vitamin, different conditions must be applied to the HPLC system to assess each vitamin. Kim published a paper in which ion pairing chromatography was used to monitor six different vitamins (nicotinic acid, nicotinamide, folic acid, and pyridoxine) in the feed [383]. Sodium hexanosulfate was used to with this approach; all six vitamins can be quantified using the same chromatographic run (using the same wavelength and column for all species) (Figure 12).

A usual problem that arises with the analysis of this compounds is the fact that pH changes affect their chemical behavior drastically both during extraction and chromatographic separation. The author circumvented this issue using a mobile phase and sample extraction solution spiked with 0.1 mL/100 mL acetic acid, maintaining all species protonated. Midttun and coworkers described an extensive two-phase analysis based on an LC-MS/MS (to determine fat soluble and water soluble). In the chloroform/isooctane phase all-*trans* retinol, 25-hydroxyvitamin D_2_, 25-hydroxyvitamin D_3_, α-tocopherol, γ-tocopherol, and phylloquinone were retained. The hydrophilic phase (in which water-soluble vitamins were found), was mixed with ethanol, water, pyridine, and methyl chloroformate as a derivatizing agent. In this assay there can be a third phase (i.e., the methyl chloroformate fraction) that it is reserved for gas chromatography analysis of amino acids [384]. As an excellent example of hydrosoluble vitamins analysis using LC-MS/MS in the food industry, is the determination of 15 compounds in beverages using a multi-mode column (SM-C_18_ column, 150 × 2.0 mm, 3 μm; Imtakt Co., Kyoto, Japan), which provided reverse-phase, anion- and cation-exchange capacities, and therefore improved the retention of highly polar analytes such as water-soluble vitamins. The use of this column removes the need for an ion pair reagent in the mobile phase [385]. Finally, we encourage the reader toward a recent and ample review regarding fat- and hydrosoluble vitamins, respectively [386,387].

#### 4.5.3. Method Application Experience

In our experience in the development of a methodology for the determination of fat-soluble vitamins in food matrices, the most challenging part has been the sample pretreatment. As mentioned above, several factors have to be considered. For the species retinyl acetate and palmitate, we chose to use a direct extraction to avoid decomposition by the saponification process. Extraction can be performed with hexane, ethyl acetate or chloroform; as the last solvent is far easier to eliminate, during concentration steps, is considered the most suitable option. Isopropanol is a useful solvent for reconstitution.

**Figure 13:**
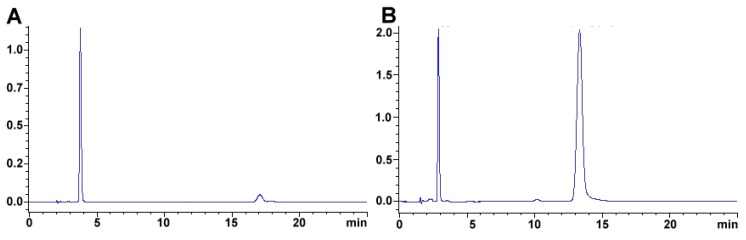
Chromatographs for vitamin A standards mixtures separated with C_8_ column at 325 nm and 50 °C, of (**A**) retinyl acetate (3.11 min) and retinyl palmitate (17.63 min) using MeOH/H_2_O (90:10) and (**B**) retinyl acetate (2.89 min) and retinyl palmitate (13.30 min) using MeOH/2-propanol/acetonitrile (95:1.5:3.5).

This method applies to dry matrices as flour, bakery products, freeze-dried pulps or fortified sugar. In the case of dairy products, we highly recommend the use of dichloromethane/ethanol. Separation is carried out using an HPLC-DAD set at 325 nm and a Zorbax Eclipse XDB-C_8_ (150 × 4.6 mm, 5 µm) column at 50 °C. Shifting the solvent system form a MeOH/H_2_O (90:10) to MeOH/2-propanol/ACN (95:1.5:3.5) saves up to 5 min of chromatographic run time and better peak shape, for the palmitate, is obtained (Figure 13A,B).

Monitoring the analytes at 295 nm, using MeOH/H_2_O (90:10) as solvents at a flow rate of 0.5 mL min^−1^, tocopherols can be successfully separated (i.e., retention times for δ/γ/α-tocopherol 5.35, 6.03 and 8.59 min, respectively; *R_s_* 1.97). In samples with relatively high-fat content, saponification is necessary to eliminate interferences that usually share similar retention times that those for α-tocopherol. Food and feed samples can be fortified or can naturally contain both, vitamin D_2_ and D_3_. Therefore, for a method to be suitable, simultaneous detection of both analytes is a must. Under the conditions above, C_8_ stationary phases are incapable of resolving both species.

**Figure 14:**
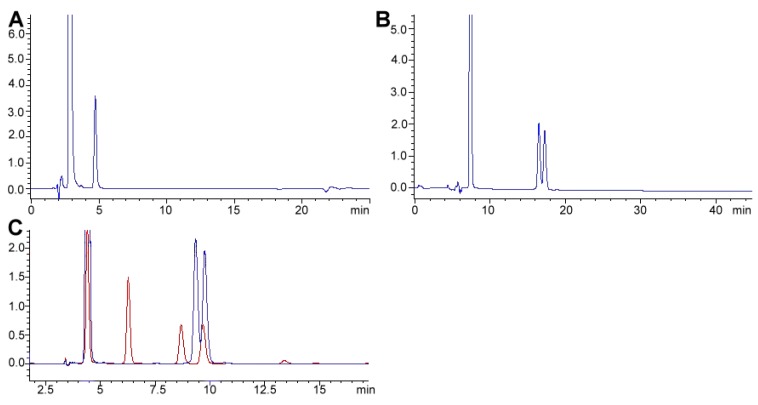
Separation for vitamin D_2_+D_3_ standards at 264 nm using (**A**) a C_8_ column and (**B**) a C_18_ column D_2_ (16.47 min) y D_3_ (17.24 min). Analysis performed at 30 °C using MeOH/2-propanol/ACN (90:3:7) (**C**) Superposed chromatograms for vitamin D_2_ + D_3_ (blue line) and δ/γ/α-tocopherol standards using a C_18_ column and MeOH/H_2_O (90:10), 30 °C.

A C_18_ column heated at 30 °C and a MeOH/2-propanol/ACN (90:3:7) solvent system, with a flow set at 0.3 mL min^−1^, can achieve a resolution of 1.25 (Figure 14A,B). Though a mobile phase composed MeOH and H_2_O is highly desirable, a drawback using this solvent system in complex matrices is that α-tocopherol can be interference for the identification of vitamin D_3_ and vice versa (Figure 14C). A solvent gradient, a column with longer alkyl chains (e.g., C_30_) or the use an MS detector may be employed to solve this issue.

## 5. Conclusions

LC is a powerful and versatile tool for food and feeds analysis, food and feed matrices are complex mixtures that on occasion present to the researcher difficulties as analytes of interest must be extracted and purified before injecting into the LC system. Advantages that chromatography provides when applied to food or feed analysis include sensitivity (determination of trace amounts, especially important in the case of contaminants, residues, controlled or undesired substances), automation and high throughput (reducing time and user dependence in laboratories with considerable workloads), simultaneous determination of multiple analytes. Food and feed chemists must make an effort to develop methods that provide a faster response and with few possible numbers of steps. Several current methods that are based on other step-full or less automated, or specific techniques can be reinvented, transformed and transplanted to LC analysis to improve sensitivity, specificity, and selectivity (For example, exchanging a spectrophotometric-based piperine analysis for a chromatography one). Within the myriad of alternatives, the LC approach delivers, each on its own seldom can solve a research problem, and each technique (i.e., each detector, each chromatographic column or sample treatment) has its shortcomings and limits as to which data is to provide. Usually, a multiphasic methodology is desirable to reach an appropriate conclusion. Hence, LC methods are more useful when are tailored to fit a purpose. Nowadays, mass spectrometry coupled liquid chromatography is an almost widespread technique that can provide molecule confirmation, ease trace analysis and allows the assay of multiple analytes simultaneously. Not only is a versatile tool for routine analysis but, research-wise, it provides more information about the target molecules and opens a valuable doorway toward a myriad of applications in food analysis including metabolomics, proteomics, and parvomics. Notwithstanding, as demonstrated above, traditional detectors are still the most commonly available in most laboratories. With a proper sample pretreatment, some traditional-detector-based methods are, regarding analytical performance, comparable to those based on MS.

## Figures and Tables

**Table 1:** Typical food and feed analytes assayed using HPLC (High-Performance Liquid-Chromatography).

**Additives**	
**Analyte Category Examples**	**Relevance in Feed and Food Quality**
**Acidulants**	Used in beverage, food, and feed production, are part of the primary metabolism, are often produced by fermentation. Acidic additives serve as buffers to regulate acidity, antioxidants, preservatives, flavor enhancers, and sequestrants. Related to beneficial effects on animal health and growth performance as feed additives.
Acetic acid, lactic acid, and citric acid [13].	
**Antioxidants**	Lipid and protein oxidation can impact meat quality, nutrition, safety, and organoleptic properties. Antioxidants are added during animal production and meat processing to enhance the nutritional and health benefits of meat and minimize the formation of carcinogens for the chemical safety of cooked and processed meats [14,15]. They can also be used to extend food [16] and feed [17] shelf life.
Gallic, rosemarinic, canosic, and caffeic acids, glabrene, procyanidins, quercetin, catechin α-, β-, γ-, and δ-tocopherols, Eugenol, Carnosine, Tyr-Phe-Glu, and Tyr-Ser-Thr-Ala.	
**Preservatives**	Usually, act as bacteriostatic and bactericidal agents to prevent microbial spoilage, antimicrobials not only extend shelf life, but they also enhance the product’s safety [18].
Acetates, bacteriocins, benzoates (*p*-hydroxybenzoic acid), borates, carbonates, lactates, nitrates/nitrites, parabens, propionates, sorbates, and sulfites.	
**Flavors and fragrances**	Widely used in food, beverage, feed, cosmetic, detergent, chemical and pharmaceutical formulations [19].
Alcohols, methyl ketones, 2,3-butanedione, lactone, butanoic acid, esters, isovaleric acid, pyrazines, geosmin, vanillin, benzaldehyde, terpenes.	
**Sweeteners**	Non-nutritive sweeteners have become an essential part of daily life and are in increasing demand as it is used in a wide variety of dietary and medicinal products [20]. They play a role in the reduction of table sugar [21]. In the case of artificial sweeteners, their use is controversial as they have associated with health risks [20,22] and water pollution [23]; currently, the use of natural sweeteners is supported as an alternative [24]. Sweetened products must be subject to verification to ensure the presence of the sweetener. Furthermore, sweeteners are regulated food additives [25] unless recognized as safe [26,27].
Approved as food additives: saccharin, aspartame, acesulfame potassium, sucralose, neotame, advantame. Generally regarded as safe (GRAS): Steviosides [28,29,30,31].	
**Natural Components**	
**Analyte Category Examples**	**Relevance in Feed and Food Quality**
**Inorganic ions**	Essential in both raw and processed products, related to food nutritional quality, preservation, technological processing, and safety [32].
Sulfites, sulfates, phosphate, polyphosphate, nitrate and nitrite, *N*-nitroso compounds, cyanide, bromide, bromate, chloride, chlorite, fluoride, iodide.	
**Lipids and fatty acids**	Major constituents of foods and feeds, of dietary importance as a significant source of energy. Provide essential fat-soluble nutrients. Are prone to peroxidation. Part of biological membranes.
C_1:0_ (formic/methanoic), C_2:0_ (acetic/ethanoic), C_3:0_ (propionic/propanoic), C_4:0_ (butyric/butanoic), C_6:0_ (caproic/hexanoic), C_8:0_ (caprylic/octanoic), C_10:0_ (capric/decanoic), C_12:0_ (lauric/dodecanoic), C_12:0_ (myristic/tetradecanoic), C_16:0_ (palmitic/hexadecanoic), 9c-C_16:1_ (palmitoleic/(9Z)-hexa-dec-9-enoic), stearic, oleic, ricinoleic, vaccenic, linoleic, α-linoleic, γ-linoleic, arachidic, eicosapentaenoic, behenic, erucic, docosahexaenoic, lignoceric, cholesterol [33,34,35,36,37].	
**Biogenic amines**	Nitrogen-based toxic compounds, mainly formed through decarboxylation of amino acids. Relevant for quality and safety of diverse foods such as dairy products [38], fermented goods [39] including wines [40], fishery commodities [41].
Putrescine, histamine, cadaverine.	
**Amino acids**	Part of a protein-containing diet, and as supplemented individual products. Amino acids are used in medical (parenteral) nutrition and dietary supplements [42].
The main fermentative amino acids for animal nutrition are L-lysine, L-threonine, and L-tryptophan. DL-Methionine.	
**Carbohydrates**	The most abundant feed energy in diets for some species of animals [43,44].
Glucose is the primary energy source for fetal growth, and lactose is crucial for the development of human and animal infants alike.	
**Vitamins**	Complex unrelated compounds present in minute amounts in natural foodstuffs. Essential to normal metabolism; their deficiency causes disease.
Fat-soluble: retinol (retinol (vitamin A) and retinyl acetate, and palmitate), tocopherols (α- (vitamin E), β-, γ-, and δ- and their acetates), ergocalciferol (vitamin D_2_), cholecalciferol (vitamin D_3_), phylloquinone (vitamin K_1_), menaquinone (vitamin K_2_), 7-dehydrocholesterol, β-carotene. Hydrosoluble or B complex vitamins: thiamine (B_1_), Riboflavin (B_2_), flavin mononucleotide or riboflavin-5′-phosphate, niacin/nicotinamide riboside/niacinamide (B_3_), pantothenic acid (B_5_), pyridoxine/pyridoxamine/pyridoxal (B_6_), biotin (B_7_), folates (B_9_), cobalamines (B_12_) [45].	
**Alkaloids**	Alkaloids are natural compounds with a characteristic cyclic structure and a nitrogen atom [46]. Alkaloid-containing plants are an essential part of the regular diet, present as natural constituents of several food products [46]. The most common use for alkaloid-containing plants is as stimulants increased concentrations of these compounds can be attained within the food chain as a result of food processing, as food contaminants or as food flavorings [46].
Octopamine, synephrine, tyramine, *N*-methyl-tyramine, hordenine in bitter orange products [47], morphine, codeine, thebaine, papaverine, and noscapine in poppy straw [48], caffeine and trigonelline in coffee [49], indole and oxindole alkaloids in *Uncaria* sp. [50], theobromine and caffeine in tea [51] and coffee [52], Harman alkaloids (harmane and harmine) in passion fruit [53], ergot alkaloids in animal feed (ergometrine, ergotamine, ergocornine, ergocryptine, ergocristine [54], piperine [55].	
**Residues and Contaminants**	
**Analyte Category Examples**	**Relevance in Feed and Food Quality**
**Chemotherapeutics and antiparasitic drugs**	Antibiotics are extensively utilized in productive animals with therapeutic, prophylactic, metaphylactic, growth promoting, and food effectiveness enhancing ends. These practices that have been reflected in veterinary residues in products for human consumption (meat, eggs, and milk) and is also related to directly with allergies and antimicrobial resistance.
Tetracyclines [56].	
**Mycotoxins**	Mycotoxins are practically ubiquitous contaminants, classified as teratogenic, carcinogenic and immunosuppressive, and that affects a great variety of grains, fruits and seeds, as well as eggs, dairy products, compounds feeds, and other feed ingredients [57].
Aflatoxins [58].	
**Pesticides**	Used for crop protection and to treat infestations in livestock. Their poor use results in contamination of the environment and the food itself, impacting human health. Residues usually found in vegetables, fruits, honey, fish, eggs, milk, and meat, serving as potential sources of contamination to consumers [59,60,61].
Atrazine, glyphosate, aminomethylphosphonic acid, phenoxy herbicides.	

**Table 2:** General conditions required for each mode of chromatography.

Type of Liquid Chromatography	Micro	Semi-Micro	Conventional	Semi-Preparative	Preparative	Process
Column internal diameter, mm	0.3 < *x* ≤ 1.0	1.0 < *x* ≤ 3.0	4.0 < *x* ≤ 8.0	8.0 < *x* ≤ 20.0	20.0 < *x* ≤ 50.0	*x* > 50.0
Eluent flow rate, mL min^−1^	0.001 < *x* ≤ 0.1	0.1 < *x* ≤ 0.4	0.4 < *x* ≤ 2.0	2.0 < *x* ≤ 10.0	10.0 < *x* ≤ 150.0	*x* > 150.0

**Table 3:** Characteristics of the most common detectors used in liquid chromatography.

Detector Type Range of Applications, Attributes, and Minimal Detectable Quantity Limitations	Applications in Feed and Food Quality
***Non-Destructive Detectors***	
**Photodiode-Array (PDA)/Variable wavelength (VW)/UV-vis**	Sulfonated azo dyes in beverages, hard candy and fish roe samples [62], purity of caffeine reference material [63], sulfamethazine and trimethoprim in liquid feed premixes [64], nitrofurans animal feed [65].
Selective; universal at low wavelengths, 3D spectra comparison can determine peak purity, can detect nanograms. Chromophore must be presesent, solvents transparent to the wavelength used must be provided.	
**Fluorescence (FL)**	
Very selective and specific; monitors two wavelengths simultaneously, 3D fluorescent spectra, fluorescent fingerprinting/fluorescence pattern analysis [66] gradients do not affect baseline significantly, can detect low picograms. Fluorophore must be present, derivative formation and quenching are often needed.	Sulfonamides [67] and fluoroquinolones [68] in animal feed, aflatoxins in agricultural food crops [69] and milk [70].
**Electrochemical (EC)**	Macrolide antibiotics in animal feeds [71], vitamin C in oranges and apples [72].
Very selective; oxidation or reduction must be possible, can detect from femtograms to nanograms. Conductive mobile phase, susceptible to background noise and electrode degradation.	
**Refractive index (RI)**	Inulin in chicory roots [73], total carbohydrates in wine and wine-like beverages [74].
Universal (All compounds affect refractive properties) and versatile; solvent compatible, relatively simple, can detect micrograms. Gradient incompatible, high S/N ratio when the pump is mixing two or more solvents, susceptible to temperature and flow variation.	
**Conductivity**	Choline, and trimethylamine in feed additives [75], L-carnitine, choline, and metal ions in infant formula [76].
Selective; an ionic form of compound necessary can detect low pictograms. Suppression of mobile phase background conductivity, special equipment required.	
***Destructive detectors***	
**Mass spectrometry**	Analysis of acrylamide in food [77], tiamulin, trimethoprim, tylosin, sulfadiazine, and sulfamethazine residues in medicated feed [78], multiclass antibiotics in eggs [79], zearalenone and deoxynivalenol metabolites in milk [80], cattle feed analysis of Aspergillus clavatus mycotoxins [81], choline chloride in feed and feed premixes [82].
Selective and specific; based on a specified mass/charge ratio, ion fractionation, can detect low nanograms. Expensive, expert users are needed for equipment and data manipulation.	
**Radioactivity**	Drug metabolite identification [83].
Selective; Distribution and mass balance wide response range can detect pictograms. Large-volume flow cells can produce peak broadening and decreased the resolution.	
**Evaporative light scattering (ELS)**	*N*-acetylglucosamine and *N*-acetylgalactosamine in dairy foods [84], sucralose and related compounds [85], spectinomycin and associated substances [86].
Universal; Nonvolatile analyte nebulization, can detect in the range of nanograms. Volatile buffers required, poor reproducibility and limited dynamic range.	
**Corona-Charged aerosol [87]**	
Universal; can detect non-ultraviolet and weakly ultraviolet active compounds [88], ionized particles measured by an electrometer, can detect low nanograms. Volatile buffers required.	Erythritol, xylitol, sorbitol, mannitol, maltitol, fructose, glucose, sucrose, and maltose in food products [89], fumonisins in maize [90].

**Table 4:** Polyphenol analysis in different matrices, based on liquid chromatography, and varied approaches to determine them.

Matrix	Analytes Identified	Extraction Method	Measurement Method, Chromatographic Column	Reference
Berries	Anthocyanins (68.6%), hydroxycinnamic acids (23.9%), flavonols (4.4%)	H_2_O, membrane ultrafiltration	Preparative LC: 250 × 20 mm Eurospher 100–5 C_18_ Identification: was HPLC/DAD/ESI^±^-MS^n^, 150 × 2.1 mm, 5 μm. λ 280, 325, 360 and 520 nm	[98]
Costa Rican guava	Ellagic acid, myricetin, quercitrin, and quercetin	MeOH/H_2_O (70 mL/100 mL). Freeze-dryed pulp, mechanical dispersion	LC-TOF-ESI^±^ (m/z range 100–1000), Synergi Hydro RP 80A 250 × 4.6 mm, 4 μm.	[110]
Brazilian guava, jambolan, nance, and lúcuma	Hydrolyzable and condensed tannins, flavonols, and flavanols	Acetone/H_2_O/HCOOH (70:29:1). Freeze-dryed pulp, accelerated solvent extraction	HPLC-DAD-ESI^−^-MS^n^, Aqua RP18 150 × 2.0 mm, 3 μm.	[113]
*Perilla frutescents* (L.) Britton	Rosmarinic acid (12.7–85.3%), scutellarein-7-*O*-glucuronide (6.5–45.1%), caffeic acid, apigenin-7-*O*-diglucuronide, and apigenin-7-*O*-glucuronide	Ethanol (EtOH)/H_2_O (75 mL/100 mL). Accelerate solvent extraction (N_2_ 1200 psi 70 °C)	UPLC-PDA-ESI^−^-TOF-MS, Kintex XB C_18_ column 150 × 2.1 mm, 1.7 μm	[114]
*Solanum lycopersicum* L.	e.g., caffeic acid hexosides, homovanillic acid hexoside, and dicaffeoylquinic acid (increasing trend)	Methanol (MeOH)/H_2_O (80 mL/100 mL)	HPLC-DAD-ESI^−^-MS/MS, Zorbax 300SB-C_18_ column (2.1 × 150 mm; 5 μm)	[115]
*Rubus fruticosus* L., *Prunus spinos* L. and *Cornus mas* L.	Gallic acid (138.0–443.5 mg kg^−1^ fresh weight), rutin (13.9–22.8 mg kg^−1^ fresh weight)	HCOOH/MeOH/H_2_O (0.1/70/29.9)	LC-FLD λ_ex_ 280, 320, 322 nm λ_em_ 360 nm. Eclipse XDB C_18_ 150 × 4.6 mm	[116]
Green, herbal and fruit teas	Gallic acid, caffeic acid (+)-catechin, (–)-epicatechin, (–)-epigallocatechin, procyanidin B_1_, and procyanidin B_2_ contribute to 43.6–99.9%	95 °C for 10 min	LC-PDA/FLD scan 260–400 nm absorbance matching Zorbax Eclipse XDB-C_18_, 150 × 4.6 mm, 5 μm	[117]
Dried and candied fruit	Vanillic, ellagic, gallic, p-coumaric, chlorogenic, caffeic, ferulic, rosmarinic acids, and myricetin, quercetin, kaempferol, delphinidin, cyanidin, and pelargonidin	MeOH/H_2_O (62.5 mL/100 mL). Sonication	HPLC-DAD at 260, 280, 329, and 520 nm. Zorbax Eclipse Plus C_18_ column 150 × 4.6 mm, 3.5 µm	[118]
Pink guava	Ellagitannins, flavones, flavonols, flavanols, proanthocyanidins, dihydrochalcones, and anthocyanidins, and non-flavonoids such as phenolic acid derivatives, stilbenes, acetophenones, and benzophenones	Freeze dried pulp, MeOH/H_2_O (90:10), sonication	UHPLC-DAD-ESI^+^-MS/MS, BHE Shield RP18 150 × 2.1 mm, 1.7 μm.	[119]
Blackberry juice		Microfiltrate (tubular ceramic membrane)	HPLC-DAD-ESI^+^-IT-MS/MS Lichrosrb ODS-2 250 × 4.6 mm, 5 µm	[120]

**Table 5:** Common chromatographic conditions used for capsaicinoid analysis.

Matrix	Extraction Method	Measurement Method, Chromatographic Column	Sensitivity, mg L^−1^ or mg kg^−1^ Fruit Dry Weight	Reference
Peppers *Capsicum annuum* L.	ACN, mechanical shaking	RP-LC/MS-TOF/ESI^−^, pseudo-molecular ions [M-H]^−^ 304.2 and 306.2 m/z. IS 4,5-dimethoxybenzyl)-4-methyloctamide, 250 × 4.6, 5 µm	0.06	[128]
Natural capsaicinoid mixture (capsaicin/dihydrocapsaicin 67:33)	C_7_H_16_/EtAOc/MeOH/H_2_O (1:1:1:1)	1. Sequential centrifugal partition chromatography. 2. Nucleosil 100-5 C_18_ column (125 × 3 mm, 5 µm, UV 280 nm	Preparative chemistry	[129]
Hot chilies, green peppers, red peppers, and yellow peppers	EtOH	HPLC-UV using a wavelength of 222 nm and a Betasil C_18_ 150 × 4.6 mm, 3 μm column	0.10	[130]
Vegetable and waste oils	Immunoaffinity column, SPE loading solvent, 5 mL MeOH/H_2_O (5:95), washing solvent PBS, MeOH for elution	LC-ESI^+^-MS/MS, Hypersil Gold, 100 × 2.1 mm, 3.0 µm	0.03	[131]
Edible and crude vegetable oils	SPE C_18_, MeOH	IS capsaicin-d_3_, and dihydrocapsaicin-d_3_. RP-UPLC-ESI-MS/MS, ZORBAX Eclipse Plus C_18_ 50 × 2.1 mm, 1.8 µm)	0.5	[132]
Austrian chili peppers	ACN/H_2_O (35:65)	UV and FLD λ_ex_ 280 and λ_em_ 310 nm, UPLC^TM^ BEH C_18_ 50 × 2.1 mm, 1.7 μm	0.136	[133]
Brazilian Capsicum chinense Jacq.	MeOH sonication	UHPLC–DAD–APCI-MS/MS, Hypersil Gold C_18_ 100 × 3 mm, 1.9 μm	0.0027	[134]
South Korean red peppers	MeOH/H_2_O (95:5), 80 °C 2 h	FLD λ_ex_ 280 and λ_em_ 325 nm, Zorbax Eclipse XDB-C_18_ 75 × 3 mm, 3.5 µm)	0.06	[135]

SPE: Solid phase extraction. UPLC: Ultra-Performance Liquid-Chromatography.

**Table 6:** Summary of conditions regarding alkaloid analysis.

Matrix	Extraction Method	Measurement Method, Chromatographic Column	Sensitivity, mg L^−1^ or mg kg^−1^	Reference
**Food Samples**				
Energy drinks	Sonication for degassing	DAD 270 nm (caffeine) Nova-Pak C_18_ 150 × 3.9 mm, 5 μm, mobile phase: MeOH, NaH_2_PO_4_/hexanesulfonic acid (C_6_H_13_SO_3_H)	0.023	[148]
Energy drinks	Sonication for degassing. “Dilute and shoot”	25 mmol L^−1^ NaAOc/HAOC buffer, pH 6.0, an inertsil OctaDecylSilane-3V 250 × 4.6 mm, 5 μm, UV 230 nm	0.19	[149]
Cocoa	Defat with C_6_H_14_, Acetone/H_2_O/HAOc (70/29.5/0.5)	1. HPLC 250 × 4.60 mm, 5 μm 2. UPLC Acquity HSS T3 100 mm × 2.1, 1.8 μm	0.001 for both LCs	[150]
Cocoa-based products	Defat by mechanical dispersion with C_6_H_14_, MeOH/H_2_O (80:20)	UHPLC-Q-Orbitrap-MS/MS polyphenols (*n* = 35, ESI^−^) and alkaloids (*n* = 2, ESI^+^) Kinetex biphenyl 100 × 2.1 mm, 1.7 µm	Theobromine 0.03, caffeine 0.04	[151]
Mate beer and mate soft drinks	Sonication for degassing, ACN.	HP-TLC LiChrospher silica gel plates, fluorescence indicator and mobile phase acetone/toluene/chloroform (4:3:3) UV 274 nm	0.4	[152]
**Biological Samples**				
Human and synthetic plasma	Ultracentrifugation, 12,000 rpm	Waters Atlantis C_18_ 150 × 4.6 mm, 5 µm. Mobile phase: 15 mmol L^−1^ PBS (pH 3.5)/ACN (83:17). PDA 274 nm, IS: antipyrine	0.02	[153]
Human saliva	Chloroform/isopropanol (85:15)	Mobile phase: H_2_O/HAOc/MeOH/ACN (79:1:20:2), Kromasil 100 C_18_ 250 × 4.6 mm, 5 μm, 30 °C, UV 273 nm	0.032	[154]
Human and neonate plasma	SPE polymeric 96-well plates Strata-X™. Elution: MeOH/H_2_O/HAOc (70:29:1)	10 mmol L^−1^ PBS (pH 6.8)/ACN (gradient mode). Zorbax^®^ SB-Aq narrow bore RR 100 × 2.1 mm, 3.5 μm), 40 °C, UV 273 nm	0.1	[155]

**Table 7:** Measurement techniques meant for cholesterol in food samples.

Matrix	Extraction Method	Measurement Method, Chromatographic Column	Sensitivity, mg L^−1^ or mg kg^−1^	Reference
Egg-, dairy-and meat-based products	ACN/2-propanol	1. Supelcosil^TM^ LC-18-DB 150 × 4.6 mm, 3 μm	3	[166]
		2. Acquity UPLC^®^ BEH C_18_ 50 × 2.1 mm, 1.7 µm, UV 210 nm	0.7	
Seafood	1. In situ: KOH 2 mol L^−1^/MeOH, 80 °C, N_2_, C_6_H_14_	Vitamin E: FLD λ_ex_ 290 λ_em_ 330). Cholesterol: UV 210 nm, Supelcosil™ LCSI 75 × 3.0 mm, 3 μm, mobile phase: *n*-hexane and 1,4-dioxane (97.5:2.5) IS: tocol	Vitamin E: 0.05 Cholesterol: 10	[169]
	2. Modified Folch: MeOH/CH_2_Cl_2_ (1:2), saponification			
	3. Smedes: 2-propanol/cyclohexane (1:1.25), saponification			
Seafood	KOH 50 g/100 g/EtOH, 25 °C, 22 h, in the dark, C_6_H_14_	1. Nova Pack CN HP 300 × 3.9 mm, 4 μm, n-hexane/2-propanol (97:3), UV 210 nm (cholesterol oxides), RID (cholesterol and epoxides)	0.01	[170]
		2. Confirmation: HPLC-APCI-MS QTRAP^®^		
Dairy product	KOH 50 g/100 g/EtOH, 25 °C, 22 h, in the dark, C_6_H_14_	Restek C_18_ 150 × 6 mm, 5μm, mobile phase: ACN/2-propanol (95:5), UV 202 nm 25-hydroxy and cholesterol, 227 nm 7-ketocholesterol	11.10	[171]
Egg and dairy product and vegetable oil	1. Egg yolk and milk: pretreatment with ACN	CLC-ODS-C_8_ 150 × 6 mm, 5μm. Mobile phase: ACN/EtOH (50:50), HPLC-UV 210 nm,	0.01	[172]
	2. Liquid–liquid dispersion (DLLME) EtOH (800 µL)/CCl_4_ (35 µL).			

**Table 8:** Measurement techniques meant for mycotoxins in feed samples.

Matrix	Number of Analytes/Execution Time (min)	Extraction Method	Measurement Method, Chromatographic Column	Reference
Cassava meal, peanut cakes, cornmeal, and different sorghum varieties	25/28	MeOH/CH_3_CO_2_CH_2_CH_3_/H_2_O (70:20:10), cleanup was performed using amino SPE cartridges	LC: Symmetry RP-18 150 × 2.1 mm, 5 µm, Identification: MS/MS/ESI^+^	[177,186]
Cereals, compound feed and silages	56/50	Modified QuEChERS method	LC: Acquity UP3 HSS T3 100 × 2.1 mm, 1.8 µm, Identification: MS/MS/ESI^±^	[179]
Bovine milk	10/30	Acid acidified ACN and sodium acetate was used to separate the aqueous from the hydrophilic phase from milk	LC: Ascentis Express C_18_, 150 × 2.1 mm, 2.7 µm, Identification: MS/MS/ESI^+^	[185]
Silage	27/44	Modified QuEChERS method	LC: Gemini^®^ C_6_-Phenyl 100 × 2.0 mm, 3 μm, Identification: MS/MS/ESI^±^	[187]
Millet and Sorghum	84 and 62 respectively/Not Indicated	ACN/H_2_O/CH_3_COOH (79:20:1) mixture	LC: Gemini^®^ C_18_, 150 × 4.6 mm, 5 μm, Identification: MS/MS/ESI^±^	[188,189]

**Table 9:** Measurement techniques meant for veterinary antibiotics in food and feed samples.

Matrix	Number of Analytes/Execution Time	Extraction Method	Measurement Method, Chromatographic Column	Reference
**Recent Multiresidue and Multi-Class Analysis of Antibiotics in Feeds**				
Rendering products	40/Not Indicated	During extraction, fat was removed and clean up performed using an SPE PRiME HLB cartridge, eluate evaporated to dryness and reconstituted with ACN and formic acid	BEH C_18_ column Identification: HPLC-MS/MS/ESI^+^	[201]
Compound feed	3/Not Indicated	Formic acid/H_2_O (80:10)	Hypersil Gold HILIC (150 × 3.0 mm, 5 µm) and C_18_ (2.1 × 50 mm, 3.5 µm). Identification: HPLC-MS/MS/ESI^+^	[202]
Pig, poultry, and cattle feed	62/13	ACN/H_2_O (90:10) acidified with CH_3_COOH.	C_18_ Vensusil XBP (50 × 2.1 mm, 3.0 μm, 100 Å). Identification: HPLC-MS/MS/ESI^+^	[209]
Feed	10/Not Indicated	Acidic extraction with hydrochloric acid (0.5 mol L^−1^ aqueous solution), and purified by SPE cartridge	Acquity UPLC HSS T3 (150 × 2.1 mm, 1.7 μm). Identification: HPLC-MS/MS/ESI^±^	[213]
**Multiresidue Analysis of Antibiotics in Foods**				
Fish muscle	41/20	Extraction with ammonium formate and ACN/H_2_O (80:20)	X-SELECT C_18_ (150 × 2.1 mm, 3.5 μm) Identification: HPLC-MS/MS/ESI^±^	[205]
Shrimp	24/8	Extraction with formic acid in water and ACN	XBridge BEH C_18_ (100 × 2.1 mm, 2.5 μm). Identification HPLC-MS/MS/ESI^+^	[206]
Poultry muscle tissue and eggs	14/14	ACN extraction Centrifugation at 0 °C 45 min	Poroshell 120 ECC_18_ (50 × 3.0 mm, 2.7 μm) Identification: HPLC-MS/MS/ESI^±^ (quadrupole linear ion trap)	[207]
Honey	6/Not Indicated	Modified QuEChERS method Extraction was performed using ACN and MgSO_4_ and NaCl	ZORBAX Eclipse XDB C-18 (150 × 4.6 mm, 5 µm). Identification: HPLC-MS/MS/ESI^+^	[214]

ACN: Acetonitrile.

**Table 10:** Sample pretreatment, derivatization and measurement conditions for amino acids in feeds.

Matrix	Hydrolysis	Derivatization		Measurement Method, Chromatographic Column	Reference
**Applications in Feed and Related Matrices**					
*Spirulina* sp.	Various physical methods	2-mercaptoethanol		Licrospher 100 RP 18 125 × 4 mm, FLD λ_ex_ 360 λ_em_ 460 nm	[218]
*Spirulina* sp.	1. Total AA: HClO_4_ 8 mol L^−1^, 150 °C for 2 h, 140 °C for 4 h, 120 °C for 8 h, and 110 °C for 22 h. 2. Free AA: CCl_3_COOH, sodium deoxycholate	Triethylendiamine (TEA), phenylisothiocianate (separation of protonated species)		Supelcosil LC_18_-DB 250 × 4.6 mm, 5 µm. Gradient 0.7 mol L^−1^ acetate buffer pH 6.4/TEA, H_2_O and ACN/H_2_O (80:20). UV λ 254 nm	[219]
*Spirulina* sp.	Pyrogallol, HCl 8.3 mol L^−1^ 70–80 °C 2 h, IS triundecanoin	o-phtaldialdehyde (OPA)		Zorbax AAA at 40 °C 40 mmol L^−1^ NaH_2_PO_4_ pH 7.8, ACN/MeOH/H_2_O (45:45:10), 2.0 mL min^−1^, UV λ 338 nm	[220]
Plants	Soncation, EZ:faast^TM^ Free Amino Acid Kit	propyl chloroformate		UHPLC EZ:faast^TM^ 4u AAA-MS, 250 × 2.0 mm, 3 µm. IS: homoarginine, methionine-d_3_, and homophenylalanine	[221]
Chamomile flowers	Free amino acids: Sonication	AccQ Fluor, 55 °C		Shimpack column (250 × 4.6 mm, 5 μm) FLD λ_ex_ 250 λ_em_ 395 nm	[222]
Rapeseed meal	HCl 6 mol L^−1^, 110 °C 23 h	Ninhydrin		Ion exchange chromatography, Vis 570 nm (Pro 440 nm), IS: Norleucine	[223]
Feed ingredients	HCl 6 mol L^−1^ 0.1 g/100 mL phenol, 150 °C 6 h, Reacti-Therm^TM^	Borate buffer pH 10, OPA (primary-) and FMOC (secondary amines)		Zorbax Eclipse-AAA 40 °C, λ_ex_ 262 λ_em_ 338 nm	[224]
Feed	6 mol L^−1^ HCl 110 °C 16–23 h, peformic acid, HBr,	AQC, borate buffer		AccQ-Tag Ultra C-18 100 × 2.1 mm, 1.7 µm). UPLC PDA 260 nm, IS: DL-2-aminobutyric acid	[225]
Fish tissue	6 mol L^−1^ HCl 110 °C closed vessel 24 h	AccQ-Fluor Reagent (AQC in 0.2 mol L^−1^ borate buffer pH 8.8)		FLD λ_ex_ 250 λ_em_ 395 nm. RP C_18_	[217]
**Selected Applications**					
Matrix	Hydrolysis and treatment		Measurement method, chromatographic column		Reference
Lipoprotein	Sodium dodecyl sulfate (SDS), enzymatic digestion (e.g., pronase E, muramidase)		UPLC BEH C_18_ 50 × 2.1 mm, 1.7 µm, 130 Å, UV 202–208 nm. Phosphate buffer 50 mmol L^−1^ pH 4.35/sodium azide and Phosphate buffer 75 mmol L^−1^ pH 4.95/MeOH (85:15)		[226]
Peptidoglycan	SDS, sonication, DNAse, RNAse, and trypsin. HCl for teichoic acids. Hydrolases (mutanolysin)		CF_3_COOH/MeOH, UPLC-TOF/MS-ESI^+^ CSH C_18_ 100 × 2.1 mm, 1.7 µm, UV 210 nm		[227]
Cocoa beans	Fermentation, HCl 0.1 mol L^−1^, mechanical dispersion, ethyl ether		UPLC-ESI^+^-MS Acquity UPLC BEH C_18_ 150 × 2.1 mm, 1.7 µm and LC/ESI^+^-MS/MS Aeris Peptide XB-C_18_ 150 × 2.1 mm, 3µm		[228]
Olive seeds	n-hexane defat, Tris/HCl pH 7.5, SDS, dithiothreitol, high-intensity focus ultrasound, acetone precipitation, alcalase hydrolysis		RP-HPLC Jupiter Proteo 250 × 10 mm, 4 µm FLD λ_ex_ 280 λ_em_ = 360 nm RP-HPLC-ESI^+^-QTOF-MS, Ascentis Express Peptide ES-C_18_ 100 × 2.1 mm, 2.7 μm		[229]

**Table 11:** Measurement techniques meant for triphenylmethane dyes.

Matrix	Extraction Method	Measurement Method, Chromatographic Column	Reference
Fresh fish muscles	Extraction with 0.1 mol L^−1^ NH_4_O_2_C_2_H_3_ buffer, pH 4.5, HAH solution 0.25 g mL^−1^, 1 mol L^−1^ p-TSA solution and can	LC: Cloversil-C_18_ 250 × 4.6 mm, 5 µm. Identification: MS/MS/ESI^+^	[243]
Channel Catfish muscle	Extraction with McIlvaine buffer, TSA, and TMPD. Oasis MCX SPE columns	LC: Prodigy ODS-3 C_18_ 150 × 4.6 mm, 3 μm. Identification: MS/MS/ESI^+^	[244]
Aquaculture water	Not indicated	LC: Phenomenex C_18_ 140, 250 × 4.6 mm, 5 μm. Identification: UV 558 nm (malachite green and crystal violet), FLD λ_ex_ 265, λ_em_ 360 nm (leuco forms)	[245]
Processed fish products	Extraction with ammonium acetate buffer, HAH solution, p-TsOH solution, and can	LC: 250 × 4.6 mm, 5 μm Capcell PAK C_18_ Identification: MS/MS/ESI^+^	[246]
Fish tissue	Modified QuEChERS Extraction: NH_4_O_2_CH and can	LC: XCharge C_18_ column	[247]
Salmon	Extraction C_2_H_3_O_2_^−^ buffer, p-TSA solution, hydroxylamine and can	YMC phenyl 3-4-5 50 × 4.0 mm, 3 µm Identification: LC-MS/ESI^+^/APCI	[248]
Fish feed	Extraction with ACN/CH_3_OH/CH_3_COOH	Chromolith^®^ Performance RP-18e (100 × 4.6 mm) Identification: MS/MS/ESI^+^	[249]

**Table 12:** Common chromatographic approaches for the determination of nitrate and nitrite ion.

Matrix	Mobile Phase Composition		Measurement Method, Chromatographic Column	Reference
**Ion Exchange Chromatography**				
Leafy greens	10 g L^−1^ of KH_2_PO_4_, pH 3.0		Waters IC-PAK HC anion exchanger (150 × 4.6 mm), UV λ 214 nm	[263]
Baby foods	Phosphate 5 mmol L^−1^ (pH 6.5)		Waters IC-PAK HC anion exchanger (150 × 4.6 mm), UV λ 214 nm	[264]
Vegetables	Phosphate 5 mmol L^−1^ (pH 6.5)		Waters IC-PAK HC anion exchanger 150 × 4.6 mm, 10 µm, UV λ 214 nm	[265]
**Reverse Phase Chromatography**				
Matrix	Ion pair reagent	Mobile phase composition	Measurement method, chromatographic column	Reference
Cured meat and vegetables	Tetrabutyl ammonium (TBA)	MeOH:H_2_O (75:25)	Phenomenex C_18_ 110 Å Gemini 250 × 4.6 mm, 5 µm. PDA λ 214 nm	[266]
Vegetables	TBA, Greiss reagent	Gradient MeOH/ACN/H_2_O	X Bridge C_18_, 50 × 2.1 mm, 2.5 µm. UV-Vis λ 222 (nitrate) and 520 nm (nitrite)	[267]
Cured meats	3 mmol L^−1^ TBA	ACN/2 mmol L^−1^ HPO_4_^2−^ pH 4	RP-thermophenyl hexyl, 150 × 4.6 mm, 3 μm, UV λ 205 nm	[270]
Vegetables	0.1 mol L^−1^ octyl ammonium salt	OA buffer pH 7.0/MeOH (70:30)	Phenomenex Luna C_18_ 250 × 4.6 mm, 5 μm, UV λ 213 nm	[271]
Dried vegetables and water	Triethylamine (TEA)	C_6_H_13_SO_3_H, H_2_PO_4_^−^, TEA pH 3.0/MeOH (80:20)	C_13_ 250 × 4.6 mm, 5 μm, UV λ 222 nm.	[272]
Ham	n-octylamine/TBA	0.01 mol L^−1^ n-octylamine/5 mmol L^−1^ TBA pH 6.5	Acclaim^TM^ Polar Advantage and C_18_ Thermo Scientific™, HyPURITY™, 250 × 4.6 mm, 5 μm	[273]

**Table 13:** Common chromatographic approaches for the determination of carotenoids.

Matrix	Extraction Method	Measurement Method, Chromatographic Column	Reference
Camu–camu (Myrciaria dubia (Kunth) Macvaugh)	Extracted from the crushed peel with acetone transferred to petroleum ether/diethyl ether and saponified with 10% KOH methanolic	HPLC-PDA Quantitative: C_18_ Nova-Pak ODS 300 × 3.9 mm, 4 µm set at 29 °C, mobile phase ACN/H_2_O/ethyl acetate For Qualitative: C_30_ YMC Carotenoid 250 × 4.6 mm, 3 µm at 33 °C. Mobile phase MeOH/MTBE (methyl tert-butyl ether)	[282]
Algae species, Chlorella vulgaris, and Scenedesmus regularis	Extraction with n-hexane–EtOH–acetone–toluene (10:6:7:7) 1 h, Saponification: 40 g/100 mL methanolic KOH at 25 °C in the dark for 16 h	PDA, YMC Carotenoid (250 × 4.6 mm, 5 µm, MeOH/ACN/H_2_O (84:14:2) and CH_2_Cl_2_ gradient UV λ 450 nm	[284]
Tissues of a species of colored bird (Taeniopygia guttata)	Plasma and liver extract: n-hexane:MTBE (1:1) Adipose tissue, retina, beak, legs: Saponification 0.02 mol L^−1^ methanolic KOH for 6 h, organic solvent extraction	PDA, YMC Carotenoid 250 × 4.6 mm, 5 µm, MeOH:ACN:CH_2_Cl_2_ linear gradient)	[285]
Taiwanese sweet potatoes (*Ipomoea batatas* (L.) Lam.)	Extraction with hexane/acetone/EtOH (2/1/1) containing MgCO_3_ and BHT (butylated hydroxytoluene) by 0.5 h, Saponification with 40 g/100 mL methanolic KOH for 3 h under nitrogen gas at 25 °C	PDA, YMC Carotenoid 250 × 4.6 mm, 5 µm, at 25 °C, MeOH–ACN–H_2_O (84:14:2) and CH_2_Cl_2_, UV λ 450 nm	[287]
Mashed orange-fleshed sweet potato	Extraction with acetone, THF, and THF:MeOH (1:1)	PDA Phenomenex LUNA C_18_ ODS, 250 × 4.6 mm, 5 µm, ACN:THF:MeOH: 1 g/100 mL NH_4_C_2_H_3_O_2_ (68:22:7:3) at room temperature and 450 nm	[288]
Selected vegetables	Extraction with THF and MeOH (1:1), petroleum ether containing 0.1 g/100 mL BHT and 50 mL 10 g/100 mL NaCl	HPLC–APCI^±^–MS, Phenomenex Luna Si C_18_ column (250 × 2 mm, 5 μm), ACN 0.1 g/100 mL/MeOH (0.05 mol L^−1^ NH_4_C_2_H_3_O_2_, 0.05 mL/100 mL TEA)/CHCl_3_ (0.1 g/100 mL BHT)/n-heptane (0.1 g/100 mL BHT), ambient temperature	[289]
Fresh and Processed Fruits and Vegetables	Extraction under subdued yellow light. 50:50 acetone/hexane Saponification: saturated methanolic KOH SPE Alumina N Sep Pak	UV, YMC Carotenoid 250 × 4.6 mm, 5 µm, mobile phase 89:11 MeOH/MTBE	[290]
Papaya (*Carica papaya* L., cv. *Maradol*)	Freeze-dried papaya homogenized in hexane: CH_2_Cl_2_ (1:1). Organic phase was separated and saponified with methanolic KOH 40 g/100 mL (1:1) for 1 h at 50 °C	HPLC-DAD-MS/MS-ESI^+^, C_30_ YMC Carotenoid 250 × 4.6 mm, 3 µm, at 15 °C. The mobile phase MeOH and MTBE	[291]
Papaya (*Carica papaya* L.)	MeOH ethyl acetate, and light petroleum (bp 40−60 °C) containing 0.1 g/L of both BHT and BHA (butylated hydroxyanisole)	DAD, YMC Carotenoid 250 × 3.0 mm, 3 µm at 25 °C. Mobile phase MeOH/MTBE/H_2_O (91:5:4) and MeOH/MTBE/H_2_O (6:90:4)	[292]
Yellow and red nance fruits (Byrsonima crassifolia (L.) Kunth)	Sample with CaCO_3_, NaCl solution (30 g/100 mL, were extracted MeOH/ethyl acetate/light petroleum 1:1:1 Saponification: methanolic KOH 30 g/100 mL stirring for 23 h	HPLC-PDA-MS/ESI^+^, C_30_ YMC Carotenoid 250 × 3.0 mm, 3 µm, MeOH/H_2_O/aqueous NH_4_C_2_H_3_O_2_ 1 mol L^−1^ (90:8:2) and MTBE/methanol/aqueous NH_4_C_2_H_3_O_2_ 1 mol L^−1^ (78:20:2), gradient, at 40 °C. UV λ 450 nm	[295]
Red and Yellow Physalis (*Physalis alkekengi* L. and *P. pubescens* L.) Fruits and Calyces	Extraction: CaCO_3_, MeOH/ethyl acetate/petroleum ether (1:1:1), containing 0.1 g L^−1^ BHA and BHT., sonication	HPLC-PDA-MS/ESI^±^, C_30_ YMC Carotenoid (250 × 4.6 mm, 3 µm, mobile phase MeOH/MTBE/H_2_O (80:18:2) and MeOH/MTBE/H_2_O (8:90:2), both 0.4 g L^−1^ NH_4_C_2_H_3_O_2_.Gradient.	[297]

**Table 14:** Different methods and stationary phases to assess carbohydrates in food matrixes.

Matrix	Sample Pretreatment, Extraction	Mobile Phase Composition	Measurement Method, Chromatographic Column	Reference
**Amine-Based Columns**				
Molasses	1. SPE Sep-Pak C_18_ 2. Microporous resin discoloration (Seplite LX), 30 °C	ACN:H_2_O (75:25)	RID, IS: maltose 1. Zorbax Carbohydrate 2. Ultimate^TM^ XB-NH_2_; both 250 × 4.6 mm, 5 µm	[307]
Tomato	Filtration, ACN/H_2_O (45:55), SPE Chromabond NH_2_	ACN:H_2_O (80:20)	Nucleodur 100-5 NH_2_ 125 × 4 mm, RID, IS: lactose	[306]
**Amide-Based Columns**				
Confectionery, chocolate products, snacks	Defat (when applicable), H_2_O 80 °C (+EtOH for chocolate products)	gradient ACN/H_2_O + 0.05 mL/100 mL ethanol- and triethyl- amine	UPLC-ELSD, Acquity BEH Amide (50, 100, 150) × 2.1, 1.7 µm, 85 °C	[308]
Apple Juice	Filtration	H_2_O	RID, Sugar Pak^TM^ 300 × 6.5 mm, 10 µm, 80 °C	[309]
**Ligand-Based Columns**				
Tubers	1. H_2_O 92 °C 2. Reflux MeOH/H_2_O (50:50) 3. Activated Charcoal/MeOH 4. 2x·MeOH/H_2_0 50:50 SPE Bond-Elut C_18_	10 mmol L^−1^ H_2_SO_4_	RID, UHPLC Aminex HPX 87H 300 × 7.8 mm, 9 µm, 18 °C	[310]
Foods	Liquids: H_2_O/EtOH (50:50) Solids: H_2_O 65 °C, sonication, +EtOH Fat-/Protein-rich: H_2_O/EtOH (20:80), sonication	1. H_2_O 2. ACN:H_2_O (9:1)	1. HPLC-RID Ultron PS-80P 300 × 6.5 mm, 10 µm, 50 °C 2. LC-MS-ESI^±^ Unison UK-Amino 150 × 2.0 mm, 3 µm	[311]
**Derivatization-Based Approaches**				
Fruit tree buds	MeOH extraction, benzyl alcohol/NaOH 8 mol L^−1^, SPE C_18_	Gradient ACN/H_2_O	PDA λ 228 and 248 nm, Shim-pack C_18_ 250 × 4.6 mm, 5 µm	[312]
Foods	2,3-naphtalenediamine, iodine, HAOc	NH_4_O_2_CH_3_ 50 mmol L^−1^ pH 5.0 in ACN/MeOH (70:30)	RID (sucrose/fructose)/UV λ 310, FLD λ_ex_ 320 λ_em_ 360 nm, C_18_ 250 × 4.6 mm	[313]
		**Normal Phase**		
Glycine max (L.) Merr	H_2_O 55 °C, ACN	H_2_O/ACN + Acetone (75:25)	ELSD, Prevail^TM^ Carbohydrate ES 250 × 4.6 mm, 5 µm	[314]
**Complex Carbohydrates (e.g., Inulin and Fructans)**				
Plants and feed materials	H_2_O	H_2_O or H_2_SO_4_ 0.01 mol L^−1^	1. Knauer Eurokat Pb 2. Nucleosil CHO 620 3. Nucleosil CHO 682 (Pb) 4. Biorad Aminex HPX-87C. All columns 300 × 7.8 mm, RID	[315]
Wheat	HCl 60 mmol L^−1^ 70 °C, quenching Na_2_CO_3_	90 mmol L^−1^ NaOH	Carbopac-PA-100 250 × 4.0 mm, PAD	[316]
Fungus sucrose fermentation	Filtration	ACN/0.04 g/100 mL NH_4_OH (70:30)	RID, Knauer Eurospher 100-5 NH_2_ Vertex 25 × 4.6 mm	[317]
Starch from feeds	heat stable amylase and amyloglucosidase	ACN/H_2_O (80:20)	RID, Zorbax Carbohydrate 150 × 4.6 mm, 5 µm	[318]
Wine	Diluted 1:9 with EtOH 70 mL/100 mL and 1-phenyl-3-methyl-5-pyrazolone derivatization	ACN/0.1 mol L^−1^ g/100 mL NH_4_C_2_H_3_O_2_ (70:30)	UV λ 245 nm, Eclipse XDB-C_18_ 250 × 4.6 mm, 5 µm	[319]
Bacterial Exopolysaccharide	Microplate polysaccharide hydrolysis 4 mol L^−1^ CF_3_COOH, 90 min at 121 °C. Derivatization 1-phenyl-3-methyl-5-pyrazolone	5 mmol L^−1^ NH_4_C_2_H_3_O_2_ pH 5.6/ACN gradient	Gravity C_18_, 100 × 2 mm, 1.8 µm, HPLC-UV-Ion trap/ESI^+^-MS	[320]

**Table 15:** Determination of organic acids and in foods and silage.

Matrix	Sample Pretreatment, Extraction	Mobile Phase Composition	Measurement Method, Chromatographic Column	Reference
**Reverse Phase-Based Columns**				
Kombucha	H_2_O 70–80 °C, filtration	20 mmol H_2_PO_4_^−^ pH 2.4/MeOH (97:3)	Luna C_18_ 250 × 4.6 mm, 5 µm 30 °C UV λ 210 nm	[337]
Fresh fruits	Juice extraction, depulping, centrifugation	50 mmol L^−1^ H_2_PO_4_^−^ pH 2.8	Hypersil Gold aQ 250 × 4.6 mm, 5 µm 10 °C UV λ 214 nm (254 ascorbic acid)	[338]
Medicinal plants infusions	(HPO_3_)_n_ extraction	3.6 mmol L^−1^ H_2_SO_4_	Sphere-Clone C_18_ 250 × 4.6 mm, 5 µm, 35 °C UV λ 215 nm (254 ascorbic acid)	[339]
Fruit juices	Filtration	0.01 mol L^−1^ KH_2_PO_4_ pH 2.6	RP-C_18_ 150 × 4.6 mm, 3 µm, UV λ 210 nm	[340]
Wine	SPE C_18_ Elution: mobile phase (for acid protonation)	0.005 mol L^−1^ H_3_PO_4_ pH 2.1, 1 mL/100 mL CAN	Lichrosorb RP-C_18_ 150 × 4.0 mm, 5 µm, UV λ 210 nm	[345]
**Ion Exchange-Based Columns**				
Sulfite pulp mill	Filtration	1. Ultrapure water 79 °C 2. Ultrapure water 68 °C 3. H_2_SO_4_ 0.005 mol L^−1^ 30 °C 4. H_2_SO_4_ 0.005 mol L^−1^ 60 °C	RID for all cases, 1. Aminex HPX-87P Pb^2+^ 300 × 7.8 mm, 9 µm 2. Transgenomic^®^ CHO-782 Pb^2+^ 300 × 7.8 mm, 7 µm 3. Bio-rad Aminex HPX-87H 300 × 7.8 mm, 9 µm 4. Shodex SH-1011 H^+^ 300 × 8.0 mm, 6 µm	[341]
Food samples	Liquid samples: filtration Solid samples: H_2_O 85 °C	0.005 mol L^−1^ H_2_SO_4_ 35 °C	Hi-Plex H 300 × 8.0 mm, 6 µm, RID	[343]
Olive fruits	Maceration in H_2_O MeOH (75:25)	0.1 g/100 mL H_3_PO_4_	Shodex RSpak KC-118 300 × 8.0 mm, UV λ 214 nm	[344]
Wines	Filtration, SPE strong anion exchange	0.065 mL/100 mL H_3_PO_4_	Aminex HPX-87H 300 × 7.8 mm, 9 µm, 65 °C, UV λ 210 nm	[346]
Fermented shrimp waste	Centrifugation, sonication, filtration	(HPO_3_)_n_ pH 2.1	SS Exil ODS 250 × 4.0 mm, 5 µm, UV λ 210 nm	[347]
Silage	H_2_O 100 °C	6 mmol L^−1^ HClO_4_	Shodex KC 811 300 × 8.0 mm, 7 µm 50 °C, UV λ 210	[348]
**Ion Exclusion-Based Analysis**				
Drinks	Filtration, heat-aided degassing	Precondition: 10 mmol L^−1^ SDS 3 h 0.3 mL min^−1^ Elution: 1.84 mmol L^−1^ H_2_SO_4_ pH 2.43	Kinetex XB-C_18_ 150 × 4.6 mm, 2.6 µm, UPLC-UV λ 210 nm	[349]

**Table 16:** Determination of fat vitamins and in foods and feeds.

Matrix	Vitamin	Extraction Method	Measurement Method, Chromatographic Column	Reference
Infant and nutritional formulas	K_1_	Lipase at 37 °C 2 h in PBS Extraction with hexane and concentrate with N_2_, reconstituted with MeOH	LC-MS/MS-ESI^+^ C_18_ 50 × 2.1 mm, 2.6 µm 40 °C. Mobile phase: H_2_O/ACN 50:50 with 0.1 mL/100 mL HCOOH and ACN/MeOH 75:25, with 2.5 mmol L^−1^ NH_4_CO_2_H, gradient system	[377]
Additives, premixes, complete feed	A, E, K_3_, D_3_	Extraction with 0.2 g/100 mL NH_3_ and EtOH, sonication 40–50 °C for 20–30 min in the dark, SPE (OASIS HLB)	HPLC-PDA C_18_ 150 × 4.6 mm, 5 μm. Mobile phase MeOH/H_2_O (98:2), UV λ 230 nm	[366]
Okra (Abelmoschus esculentus)	B_2_, B_3_, B_6_, B_12_, C, E, K, D, A, β-Carotene	Vitamin B Analyses. 0.1 mol L^−1^ H_2_SO_4_ incubation 30 min at 121 °C, pH 4.5, 2.5 mol L^−1^ NaC_2_H_3_O_2_, Takadiastase enzyme Ascorbic Acid Analyses Extraction 0.3 mol L^−1^ metaphosphoric acid and 1.4 mol L^−1^ HAOc Saponification KOH 50 g/10 g in EtOH in reflux at 50 °C for 40 min. Extraction with ether	Vitamin B Analyses. Agilent ZORBAX Eclipse Plus C_18_ 250 × 4.6 mm, 5 µm. Mobile phase MeOH/0.023 mol L^−1^ H_3_PO_4_ pH 3.54 (33:67) UV λ 270 nm at room temperature Ascorbic Acid Analyses PDA, 0.1 mol L^−1^ KC_2_H_3_O_2_ pH 4.9 and ACN/H_2_O (33:67). UV λ 254 nm at room temperature *β*-carotene: Agilent TC-C_18_ 250 × 4.6 mm, 5 µm, ACN/MeOH/ethyl acetate (88:10:2) at 453 nm. Fat-soluble vitamins Eclipse XDB-C_18_ 150 × 4.6 mm, 5 μm, MeOH at: 325, 265, 290, 244 nm	[365]
Fruits, juices, and supplements	A, D_3_, E, B_1_, B_2_, B_3_, B_5_, B_6_, B_9_, B_12_ C	Extraction with Carrez I and Carrez II solutions Cleaned by SPE (RP_18_ Bakerbond)	PDA, TSKGel ODS-100V, 150 × 4.6 mm, 5 μm at 30 °C. Gradient 0.01 mL/100 mL TFA in H_2_O and MeOH at 320, 275, 253, 290, 258, 218, 289, 360, and 262 nm	[364]
Cheeses	D_3_	Saponification with KOH (60% g/100 mL in H_2_O). Extraction: MeOH/CHCl_3_	DAD, at 228 and 266. C_18_ 200 × 4.6 mm, 5 µm ambient temperature Mobile phase: MeOH/ACN/H_2_O (49.5:49.5:1)	[378]
Dairy and soybean oil	K_1_, D_3_, E, A	Saponification: KOH/EtOH + ascorbic acid. Mechanical shaking. Extraction: hexane, evaporated at 40 °C and re-dissolved in MeOH	PDA, C_18_ 250 mm × 4.6 mm, 5 μm at 30 °C, 3.0 g/100 mL SDS and 0.02 mol L^−1^ PBS at pH 7, with 15.0 mL/100 mL butyl alcohol (organic solvent modifier). 230 and 300 nm (K_1_, D_3_ and E vitamins), 280 nm (A, E, D_3_, and K_1_)	[379]
Milk sample	K_1_, D_3_, D_2_, E, A	Extraction: hexane, concentrate with N_2_ and reconstituted with MeOH	HPLC-UV-Vis 325, 264, and 280 nm (A, D, K and E), C_18_ DBS 150 × 3 mm, 3 μm at room temperature; MeOH/H_2_O (99:1) and MeOH/THF (70:30)	[380]
Vegetables, fruits, and berries	K_1_	Extraction with 2-propanol/hexane and CHCl_3_/MeOH	HPLC-EC Vydac 201 TP54 150 × 4.6 mm, 5 μm. Mobile phase: MeOH/0.05 mol L^−1^ NaC_2_H_3_O_2_, pH 3 (96:4)	[381]
Rice	E	Saponification KOH (600 g L^−1^) in EtOH with pyrogallol for 45 min at 70 °C, mechanical shaking. Microextraction: CCl_4_/ACN (1:10)	FLD, COSMOSIL π-NAP 250 × 4.6 mm, 5 μm at 25 °C. Mobile phase: MeOH/H_2_O/ACN (80:13:7). Vitamin E isomers λ_ex_ 290 and λ_em_ 330 nm	[382]
